# Supplementary material for: Adjuvanted nanoliposomes displaying six hemagglutinins and neuraminidases as an influenza virus vaccine
Source: Cell Rep Med. 2024 Feb 23;5(3):101433. doi: 10.1016/j.xcrm.2024.101433 (PMC10982964; doi:10.1016/j.xcrm.2024.101433)
Supplement: Document S1. Figures S1‒S6 and Table S1 [file mmc1.pdf]

Cell Reports Medicine, Volume *5*

Supplemental information

Adjuvanted nanoliposomes displaying six hemagglutinins and neuraminidases as an influenza virus vaccine

Zachary R. Sia, Jayishnu Roy, Wei-Chiao Huang, Yiting Song, Shiqi Zhou, Yuan Luo, Qinzhe Li, Dominic Arpin, Hilliard L. Kutscher, Joaquin Ortega, Bruce A. Davidson, and Jonathan F. Lovell

Supporting Information: Adjuvanted Nanoliposomes Displaying Six Hemagglutinins and Neuraminidases for an Influenza Virus Vaccine

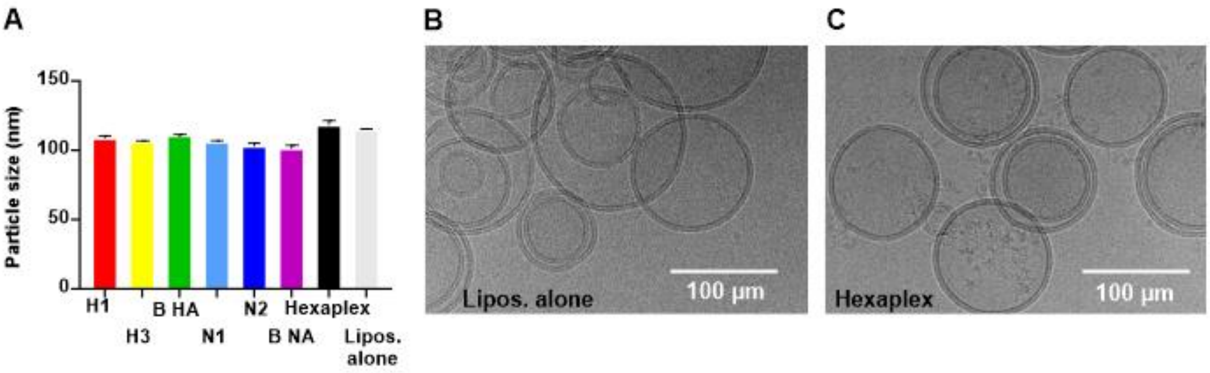

Figure S1. Particle Size Measurements. (A) Particle size is consistent between undecorated, monovalent and hexaplex liposomes. (B & C) Liposome morphology observed by cryo-TEM shows intact liposome morphology. (C) Some proteins can be observed in the objective focus on the liposome surface. Related to Figure 1. Particle size technical replicates of n = 3 were used.

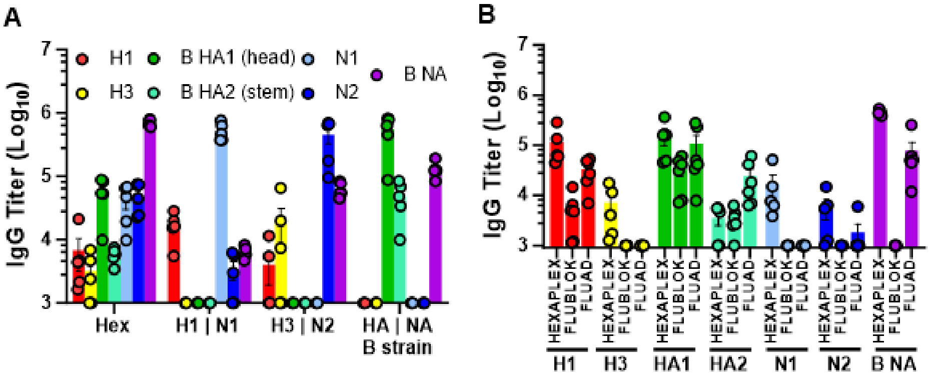

Figure S2. Antigen Cross-Reactivity Without Multimerization Domains. (A) Serum antibodies were tested against heterologous antigens without polymerization domain. These strains differed from those in figure 2; included strains were A/Wisconsin/588/2019 (H1N1), A/Kansas/14/2017 (H3N2), and B/Brisbane/60/2008. (B) Compared to Flublok and Fluad, hexaplex produced the greatest quantity of cross-reactive IgG antibodies not specific to the polymerization domains. Biological replicates of n = 5 were used. This data supplements information in the main text Figure 2 and 4.

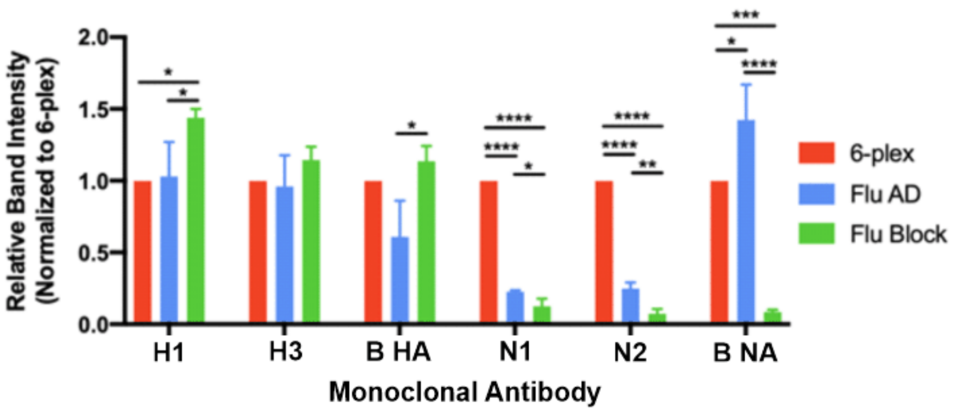

Figure S3. Slot Blot Band Quantification. Band intensity for slot blot in figure 4B, normalized to the bands presented by the hexaplex nanoliposomes. Statistical analysis was performed by one-way ANOVA with Tukey's post-hoc multiple comparisons. Markers indicate *p<0.05, **p<0.01, ***p<0.005, ****p<0.001. Related to Figure 4.

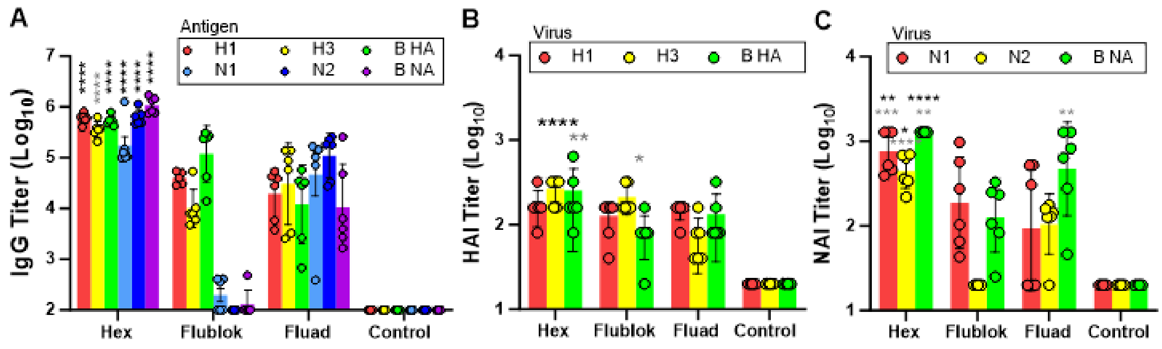

Figure S4. Additional Serological Analysis of Vaccine in Mice. Serum from mice vaccinated with hexaplex or comparator vaccines were assessed in serological assays against viruses A/California/04/2009 (H1N1), A/New York/39/2012 (H3N2), and B/Washington/02/2019 (Victoria lineage). (A) Hexaplex yielded significantly higher IgG titers than either Flublok or Fluad. (B) HAI titers were comparable among vaccines, with hexaplex yielding significant advantage over Fluad. (C) NAI was significantly higher in hexaplex vaccinated mouse serum than either Flublok or Fluad. Statistical analysis was performed with two-way ANOVA with multiple comparisons. Markers indicate *p<0.05, **p<0.01, ***p<0.005, ****p<0.001, black markers indicate significance relative to Fluad, while grey markers indicate significance relative to Flublok. Biological replicates of n = 5 were used. Related to Figure 4.

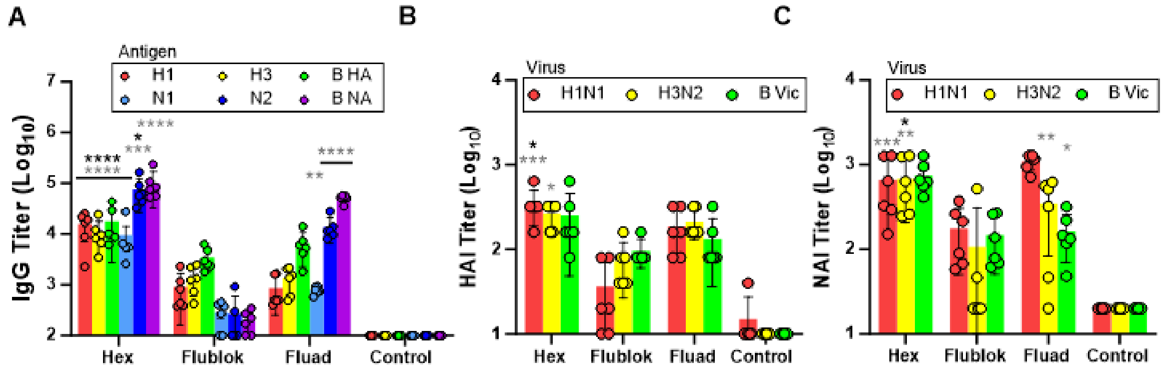

Figure S5. Serological Analysis of Hexaplex Vaccine in Ferrets. Serum from ferrets vaccinated with hexaplex, flublok, and fluad, were assessed using serological assays as previously described. (A) Hexaplex vaccine elicited highest overall specific binding antibody titers, with Fluad achieving comparable titers against B NA. (B) Hemagglutination inhibition titers were comparable between hexaplex and fluad vaccines, with hexaplex vaccine yielding significantly higher HAI against H1N1. (C) Neuraminidase inhibition was likewise comparable to Fluad, with superior NAI against B Victoria lineage virus. Asterisks indicate significance relative to hexaplex; asterisks over hexaplex bars indicate significant increase over all other groups, while asterisks over comparator bars indicates significant increase over hexaplex. Statistical analysis was performed with two-way ANOVA with multiple comparisons. Markers indicate *p<0.05, **p<0.01, ***p<0.005, ****p<0.001, black markers indicate significance relative to Fluad, while grey markers indicate significance relative to Flublok. Biological replicates of n = 6 were used. Related to Figure 5.

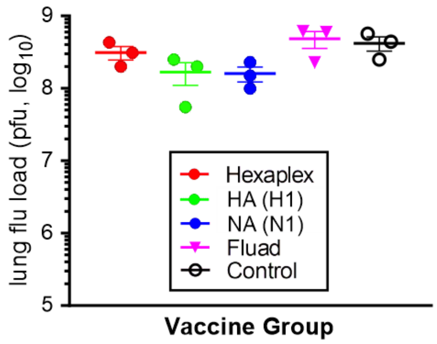

Figure S6. Lung Viral Load During Passive Transfer Challenge. Virus load in the homogenized lung tissue of mice was measured on day 4 post-infection by euthanasia and excision from 3 mice within the initial groups. Statistical analysis by one-way ANOVA identified no significant differences between groups. Biological replicates of n = 3 were used. Related to Figure 6.

% Identity with Vaccine Strains

	HA			NA		
**Virus Strain**	A/Victoria /2570/2019 (H1N1)	A/Darwin/ 6/2021 (H3N2)	B/Austria/ 1359417/2021 (Victoria)	A/Victoria/ 2570/2019 (H1N1)	A/Darwin/ 6/2021 (H3N2)	B/Austria/ 1359417/2021 (Victoria)
H1N1						
A/California/04/2009	95.05	41.70	29.26	94.46	43.07	35.46
H3N2						
A/Hong Kong/1/1968	45.39	84.28	26.97	41.93	84.22	29.85
B Victoria						
B/Malaysia/2506/2004	28.95	27.41	97.09	31.93	32.37	95.49
B Yamagata						
B/Phuket/3073/2013	28.95	28.25	92.12	32.42	28.51	94.42

Table S1. Protein Sequence Identity by Protein BLAST. Antigen sequences acquired from the GISAID database were compared by Protein BLAST multiple sequence comparison, with percentage identity of alignments for both HA and NA antigens compared by strain here. Related to Figure 3
