# Supplementary material for: Adjuvanted nanoliposomes displaying six hemagglutinins and neuraminidases as an influenza virus vaccine
Source: Cell Rep Med. 2024 Feb 23;5(3):101433. doi: 10.1016/j.xcrm.2024.101433 (PMC10982964; doi:10.1016/j.xcrm.2024.101433)
Supplement: Document S2. Article plus supplemental information [file mmc2.pdf]

Cell Reports
Medicine

Article

Adjuvanted nanoliposomes displaying six hemagglutinins and neuraminidases as an influenza virus vaccine

Graphical abstract

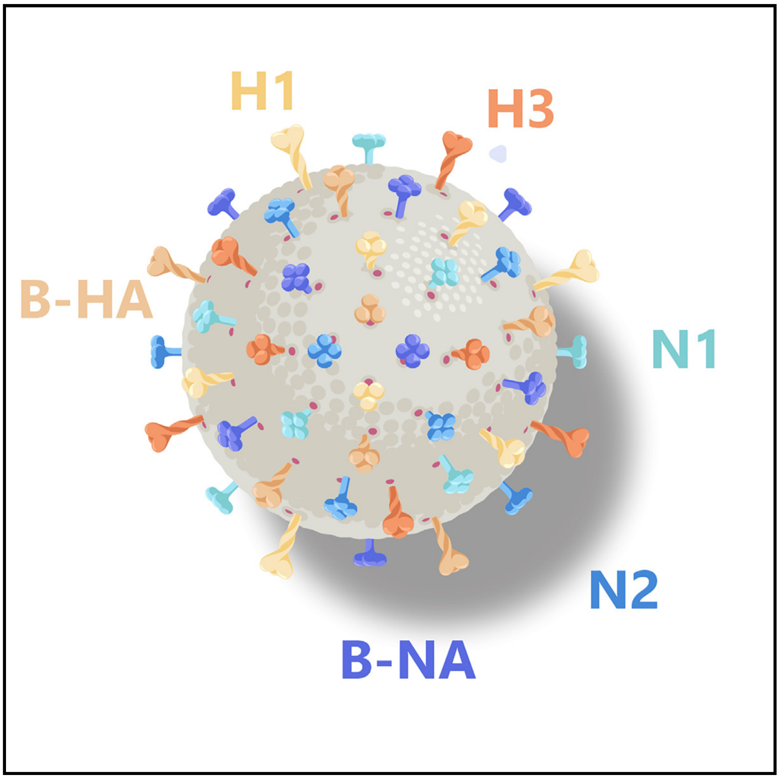

Highlights

- Recombinant hemagglutinin and neuraminidase are displayed on the surface of liposomes
- Vaccination with HA, NA, or multivalent particles protects mice from virus challenge
- Use of multivalent recombinant HA and NA nanoparticles is a promising vaccine approach

Authors

Zachary R. Sia, Jayishnu Roy, Wei-Chiao Huang, ..., Joaquin Ortega, Bruce A. Davidson, Jonathan F. Lovell

Correspondence

bdavidso@buffalo.edu (B.A.D.), jflovell@buffalo.edu (J.F.L.)

In brief

Sia et al. use an adjuvanted liposome system to display and characterize recombinant hemagglutinin and neuraminidases derived from three seasonal influenza virus strains. With vaccination, the antigens are shown to induce functional immune responses and protect against mouse-adapted virus challenge in both individual and multivalent format.

Sia et al., 2024, Cell Reports Medicine *5*, 101433
March 19, 2024 © 2024 The Author(s).
https://doi.org/10.1016/j.xcrm.2024.101433

CellPress

Cell Reports Medicine

CellPress
OPEN ACCESS

Article

Adjuvanted nanoliposomes displaying six hemagglutinins and neuraminidases as an influenza virus vaccine

Zachary R. Sia,[1] Jayishnu Roy,[1] Wei-Chiao Huang,[1,2] Yiting Song,[1] Shiqi Zhou,[1] Yuan Luo,[1] Qinzhe Li,[1] Dominic Arpin,[3] Hilliard L. Kutscher,[2,4] Joaquin Ortega,[3] Bruce A. Davidson,[5,*] and Jonathan F. Lovell[1,6,*]

[1]Department of Biomedical Engineering, University at Buffalo, State University of New York, Buffalo, NY 14260, USA
[2]POP Biotechnologies, Buffalo, NY 14228, USA
[3]Department of Anatomy and Cell Biology, McGill University, Montreal, QC H3A 0C7, Canada
[4]Department of Medicine, Jacobs School of Medicine and Biomedical Sciences, University at Buffalo, State University of New York, Buffalo, NY 14203, USA
[5]Department of Anesthesiology, University at Buffalo, State University of New York, Buffalo, NY 14203, USA
[6]Lead contact
*Correspondence: bdavidso@buffalo.edu (B.A.D.), jflovell@buffalo.edu (J.F.L.)
https://doi.org/10.1016/j.xcrm.2024.101433

SUMMARY

Inclusion of defined quantities of the two major surface proteins of influenza virus, hemagglutinin (HA) and neuraminidase (NA), could benefit seasonal influenza vaccines. Recombinant HA and NA multimeric proteins derived from three influenza serotypes, H1N1, H3N2, and type B, are surface displayed on nanoliposomes co-loaded with immunostimulatory adjuvants, generating "hexaplex" particles that are used to immunize mice. Protective immune responses to hexaplex liposomes involve functional antibody elicitation against each included antigen, comparable to vaccination with monovalent antigen particles. When compared to contemporary recombinant or adjuvanted influenza virus vaccines, hexaplex liposomes perform favorably in many areas, including antibody production, T cell activation, protection from lethal virus challenge, and protection following passive sera transfer. Based on these results, hexaplex liposomes warrant further investigation as an adjuvanted recombinant influenza vaccine formulation.

INTRODUCTION

Seasonal influenza epidemics generate a demand for safe and effective vaccines that can be produced to match evolving circulating viral strains on an annual basis. Each year, approximately 500 million vaccine doses are produced to combat seasonal influenza viruses, which still account for 27–54 million infections and 300,000–650,000 hospitalizations in the United States alone, with the burden of influenza being even greater in countries with less access to vaccines.[1,2] Recent advances in recombinant antigen production have made this method an alternative to the traditional egg-derived production methods such as inactivated virus or split-virion vaccines. However, current recombinant vaccines still possess drawbacks. Recombinant antigens may fall short of the immunogenic potential achieved by virus-derived antigens, especially in vulnerable populations such as the elderly.[3,4] These issues can be overcome in various ways, such as a greater quantity of antigens per dose or the inclusion of an immunostimulatory adjuvant component to increase effectiveness. The thrust of adjuvant development holds promise, as robust adjuvants can improve the breadth and efficacy of the immune response through its component formulation while conferring dose-sparing capability to alleviate potential bottlenecks in antigen production for commercial vaccines.[5–7] To this end, we have investigated the application of cobalt porphyrin-phospholipid (CoPoP) liposomes, which feature antigen-display functionality that converts soluble antigens into a nanoliposome-decorated format with integrated adjuvants as an adjuvant system for a next-generation recombinant vaccine, with the ultimate goal of developing a vaccine that effectively protects against all seasonal influenza viruses.

The influenza virus has two major surface antigens that serve as frequent targets for immunization: hemagglutinin (HA) and neuraminidase (NA). To date, the immunodominance of HA has made it the primary focus of commercial vaccine development, with HA inhibition (HAI) by antibodies serving as a central metric for predicted vaccine effectiveness. Dose determination by HA content serves as a major quantification metric for quality control of most current vaccines. The only currently licensed recombinant vaccine in the United States, Flublok, incorporates only HA proteins.[8] While the NA antigen has been shown to retain somewhat more conserved epitopes between strains, and antibodies against NA are known to contribute enhanced protection against infection,[9–12] the relative immunodominance of HA and widespread use of HAI as a testing metric has made NA a less frequent target for vaccine development. However, natural influenza infection results in antibodies against both HA and NA, and NA antibodies confer a breadth of protection not typically seen

© 2024 The Author(s). This is an open access article under the CC BY license (http://creativecommons.org/licenses/by/4.0/).

from HA-centric vaccines.[13,14] Providing a single, multivalent particle vaccine presenting both HA and NA antigens from multiple strains could yield an increase in protective breadth in the immune response. Multivalent ferritin-based protein nanoparticles displaying a mosaic of HAs have shown promise[15] and have inspired our approach in leveraging liposomal antigen-binding systems for multiplexed vaccines. The CoPoP liposomal platform can rapidly and flexibly incorporate both HA and NA antigens, and we have constructed a hexavalent recombinant formulation that is further enhanced by immunostimulatory vaccine adjuvants that are co-delivered in the liposome scaffold.

CoPoP liposomes provide a single-particle approach with rapid prototyping capabilities for delivering and adjuvanting multiple recombinant influenza antigens for use in vaccines. Our prior research has found that the binding properties of these liposomes result in increased efficacy over soluble antigen co-delivered with adjuvant of otherwise similar composition.[16] When incubated with His-tagged antigens, CoPoP liposomes spontaneously assemble a biostable layer of antigen decoration on their surface, resulting in enhanced immunological antigen uptake, recognition, and stimulation.[17–19] Incorporating the lipid immunostimulatory lipid molecule phosphorylated hexaacyl disaccharide (PHAD), a synthetic form of monophosphoryl lipid A (MPLA), as well as a bilayer-localized saponin adjuvant QS21 further increases immunogenicity. Both adjuvants are used in licensed vaccines and can interact synergistically in vaccine adjuvant delivery systems.[20] CoPoP liposomes with integrated adjuvant have recently undergone phase 2 and phase 3 clinical trials with presentation of the receptor-binding domain protein from SARS-CoV-2 virus.[21,22] The “hexaplex” liposomes we develop here contain six recombinant antigens representing both HA and NA components from the three recommended seasonal influenza strains H1N1, H3N2, and B Victoria lineage. They are compared against single-antigen formulations, a contemporary licensed vaccine including recombinant HA antigen vaccine (i.e., Flublok), and an adjuvanted egg-derived inactivated virus vaccine (i.e., Fluad), to determine whether a hexaplex nanoparticle solution could be an alternative for protecting against influenza infections in an annual vaccine scenario.

RESULTS

Display of HA and NA on immunogenic liposomes

Six recombinant HA and NA proteins derived from the 2022–2023 strains recommended by the World Health Organization (WHO) for Northern Hemisphere trivalent recombinant vaccines were obtained.[23] H1 and N1 were derived from A/Victoria/2570/2019 H1N1. These H1 and N1 sequences are an identical match to those from A/Wisconsin/588/2019 H1N1, which is the recommended H1N1 strain specifically for recombinant vaccines (as opposed to egg-based ones). H3 and N2 were derived from A/Darwin/6/2021 H3N2, and B HA and NA were derived from B/Austria/1359417/2021 B Victoria, as per WHO recommendation guidelines.

The concept of spontaneous, biostable binding via His-tag interaction with CoPoP in the bilayer, allowing surface integration of the antigens to form decorated liposomes, is shown in Figure 1A. Terminal His-tags, which serve not only to facilitate purification but also to anchor the antigen into the CoPoP bilayer, position the proteins on the membrane in a putatively biomimetic fashion; attached at the C terminus for HA and the N terminus for NA. The design of the recombinant full-length HA included HA1 (with native signal sequence) and HA2 domains, followed by a C-terminal fibritin trimerization foldon, then a His-tag in place of the transmembrane domain. NA included an N-terminal His-tag, followed by a VASP (vasodilator-stimulated phosphoprotein) tetramerization domain and the full-length protein (Figure 1B). With this protein design, a native-like quaternary structure and membrane orientation is assumed on the liposomes.

To achieve antigen binding to liposomes, a simple admixture step with 1-h incubation at 37°C was carried out. No further downstream purification was required and, therefore, no further quantification of vaccine components was performed after formulation. A nickel-nitrilotriacetic acid (NTA) competition assay, in which nickel beads are used to competitively bind soluble His-tagged antigens thereby revealing whether proteins have converted into membrane-bound format, determined that proteins rapidly bind with high affinity to liposomes with simple admixture of antigen and liposomes. At a 4:1 mass ratio of CoPoP to antigen, all individual antigens (i.e., H1, N1, H3, N2, B HA, and B NA) converted for the most part from soluble to liposome-bound format (Figure 1C). Incubation with the complete hexaplex formulation shows that all six proteins become liposome bound at levels consistent with or even higher than with individual incubation. The majority of proteins were incorporated even using a liposome-to-total-antigen mass ratio of 2:1, twice the antigen-to-liposome ratio of the incubation concentration used for single antigens. For subsequent studies, a CoPoP-to-antigen mass ratio of 3:1 was selected. Although overlap in molecular weight precluded identification of individual antigens in hexaplex format, all antigens bound the liposomes individually and in hexaplex format in these incubation conditions. Identical liposomes that substituted two hydrogens for cobalt and also contained PHAD and QS21 (2HPQ) did not demonstrate any protein binding, showing that the inclusion of intrabilayer cobalt was responsible for liposome display. The presence of all antigens on the surface of the liposomes was confirmed through the use of monoclonal antibodies for each component in a slot-blot assay shown in Figure 1D. The indicated monoclonal antibodies recognized each antigen without any non-specific cross-reactivity, and hexaplex liposomes were recognized by all six antibodies. In addition to confirming the presence and accessibility of the six antigens on the surface of the hexaplex liposomes, these antibodies could be useful for future studies for developing ELISA methodologies for quantification of antigenic components of the liposomes. Nanoparticle size did not significantly vary between protein contents, and aggregation was not observed following incubation (Figure S1A). Compared to naked liposomes, irregular decoration of protein antigens were observed to be bound to the surface of liposomes by cryogenic transmission electron microscopy (cryo-TEM) (Figure S1B).

Antibody response to the hexaplex vaccine

Outbred mice were immunized via intramuscular injection with 1.8 μg of total antigen per dose on day 0 and day 21, and on day 42, serum was collected for serological analysis. This

CellPress
OPEN ACCESS

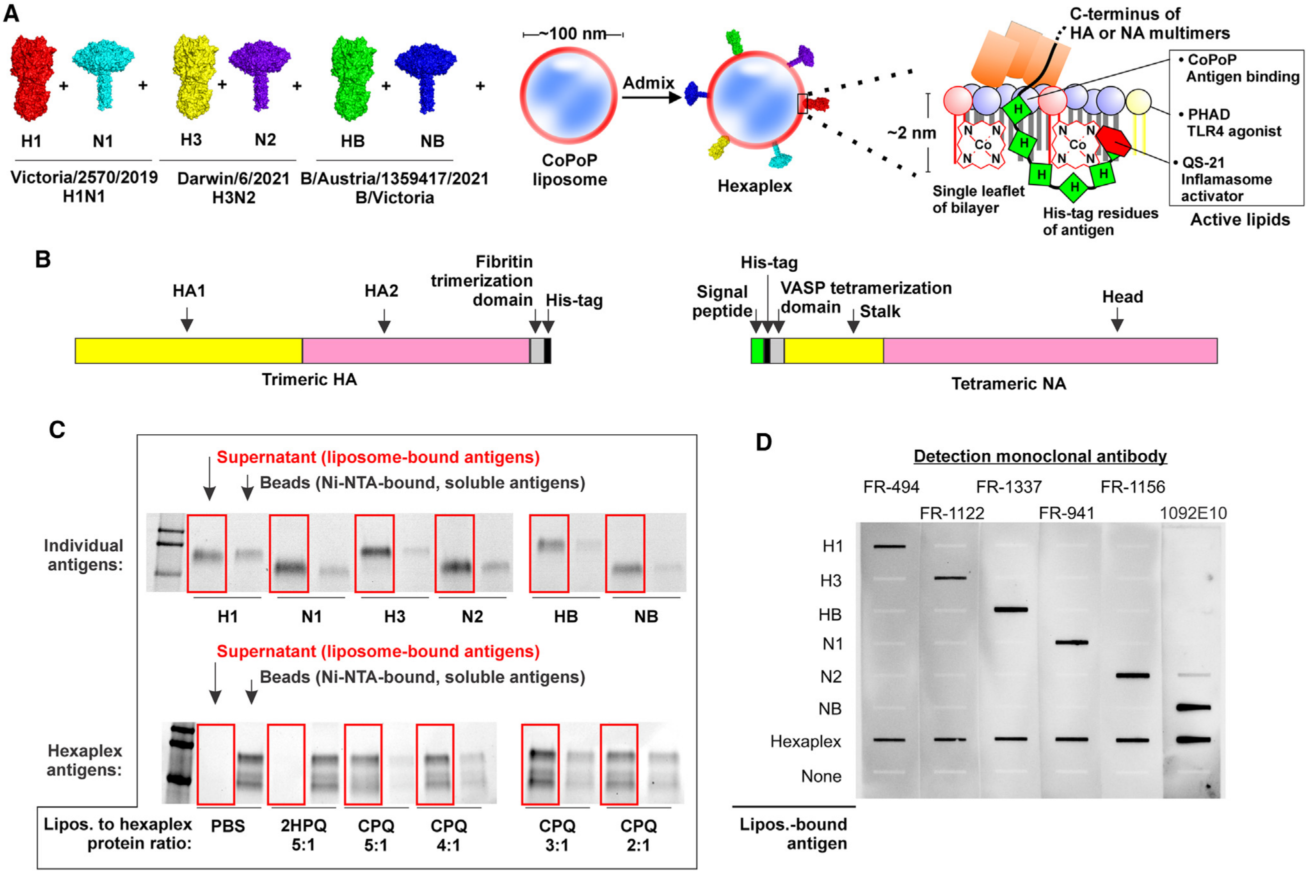

Figure 1. Design of a hexaplex liposomal vaccine

(A) Indicated influenza antigens are surface-displayed on CoPoP liposomes containing PHAD and QS21 (CPQ) via His-tag cobalt interaction to produce surface-decorated hexaplex particles.

(B) Recombinant antigens are designed with a trimerizing domain for hemagglutinin and a tetramerizing domain for neuraminidase, producing polymerized antigens that replicate the quaternary conformation and membrane orientation on influenza viruses. Nickel-NTA competition assay was performed using individual antigens as well as multiplex mixture.

(C) Each individual antigen shows high binding to liposomes in electrophoresis gel, and a solution of all six proteins shows high simultaneous binding affinity to CoPoP liposomes. Similar antigen sizes for HA and NA antigens result in bands overlapping.

(D) Monoclonal antibody reactivity of liposome-displayed antigens in monovalent or hexaplex formulations by slot-blot assay.

dosage corresponded to 5.4 μg of CoPoP, and 2.16 μg of PHAD and QS21 adjuvants in the liposome fraction. Immunoglobulin G (IgG) antibodies in mice vaccinated with single liposomal HAs showed a high degree of cross-reactivity against other hetero-subtypic HAs; the same was true between NA groups, which could be due to shared multimerization domains. Despite the cross-reactivity, each single-antigen vaccine induced the highest IgG titers against the homologous antigen, as expected. Hexaplexed nanoparticles induced a balanced response against all six antigens (Figure 2A). This held true when hexaplex-induced antibodies were compared against antigens without the shared multimerization domains, indicating a significant antibody response against the unique identity of each antigen (Figure S2A). Antibody neutralization of HA activity was assessed by *in vitro* assay of HAI, and results showed high specific neutralization in monotypic vaccine response and effective neutralization against all virus HA serotypes in hexaplex vaccine response (Figure 2B). Similarly, *in vitro* assay of neuraminidase inhibition (NAI) found that individual liposome-displayed NA antigens induced functional serum antibodies against NA (Figure 2C). In hexaplex format, antibodies were induced with NAI activity against all four viral strains assessed. However, we did not account for the effect of steric hindrance from anti-HA antibodies, which may have contributed to the observed results for the hexaplex vaccine.[24] Future NAI studies using reassortment viruses with mismatched HA types could resolve this issue.

Hexaplex vaccine protects in viral challenge

Next, inbred BALB/c mice immunized with HA, NA, or hexaplex vaccines were subjected to various mouse-adapted influenza virus challenges. The total antigen dose in all vaccines was 1.8 μg, so that the H1 group received 1.8 μg of H1 antigen, but the hexaplex group received only 0.3 μg of H1, along with equal masses of the five other proteins. The hexaplex vaccine compared

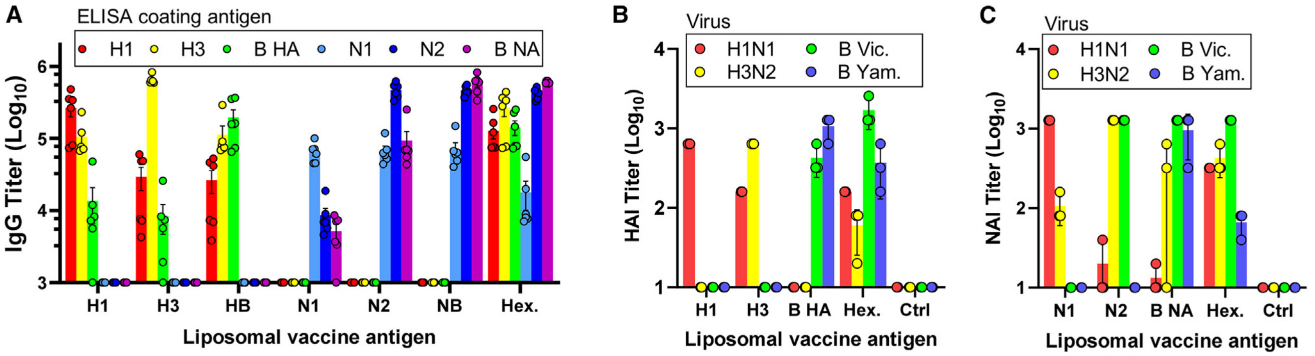

Figure 2. Functional antibodies are induced by liposome-displayed antigens

Outbred CD-1 mice were vaccinated with 1.8 μg of total antigen displayed on CoPoP liposomes on days 0 and 21, with serum collected at day 42 for analysis. IgG antibody binding titers were determined by ELISA against recombinant His-tagged antigens.

(A) Antibody quantities elicited by hexaplex mixture are comparable to single-antigen vaccines with equal total protein. Viral HA and NA inhibition assays were against mouse-adapted A/California/04/2009 H1N1, mouse-adapted A/Hong Kong/1/1968 H3N2, mouse-adapted B/Malaysia/2506/2004, and human B/Phuket/3073/2013.

(B) Hemagglutinin inhibition by antibodies assessed by red blood cell agglutination *in vitro*. Hexaplex vaccine achieves inhibition against all virus serotypes in quadrivalent formulation.

(C) Neuraminidase inhibition assessed by colorimetric enzyme-linked lectin assay *in vitro*. Biological replicates of n = 5 were used.

favorably against H1N1 in challenge (3,480 PFU, 10× median lethal dose [LD_{50}]), with the greatest weight retention (Figure 3A), lowest clinical score of symptoms (Figure 3B), and complete survival of all experimental group animals (Figure 3C). All vaccines achieved similar reduction in lung viral load against H1N1 at day 4 post challenge (Figure 3D). Interestingly, protection kinetics in H1N1 challenge appeared to be distinct for the different antigens used. Mice vaccinated with only H1 recombinant antigens and adjuvant showed minimal weight loss in the early stages but suffered a loss of weight and increase of clinical symptoms after day 6. By contrast, mice vaccinated with N1 alone appeared to be unprotected until day 5, at which point recovery of body weight occurred. Hexaplex vaccine did not exhibit time-variable protection, which suggests that H1 and N1 both contributed to protection across all phases of the infection. Against H3N2 virus (100 PFU, 2.5× LD_{50}), hexaplex vaccine was less effective in each metric than an equal total antigen dose of H3 but retained significant protection when compared to N2 or control vaccines. This is likely the result of the lower sequence identity between the vaccine and challenge H3N2 antigens, although protection was still achieved by HA despite a greater than 15% mismatch between antigen sequences, similar to the NA group mismatch (Table S1). Unlike in H1N1 infection, time-variable protection was not observed in vaccination with H3 or N2 alone. Against influenza B virus (3.2 × 10^6 PFU, 10× LD_{50}), single and hexaplex formulations were equally effective at protecting against infection. While complete protection was achieved in all B influenza vaccine groups, control mice all succumbed by day 7, indicating that high protective efficacy was a result of effective immune response rather than low viral potency.

Comparison with other adjuvants and contemporary vaccines

Outbred CD-1 mice were then immunized with various vaccine formulations to characterize performance. Mice vaccinated with the hexaplex particles exhibited higher IgG titers against each individual antigen than 2HPQ non-binding liposomes or alum colloidal adjuvant (Figure 4A). The 2HPQ vaccine was identical to the hexaplex counterpart, except that cobalt was omitted from the CoPoP lipid, thereby isolating the impact of biostable liposome display of antigens. To determine whether hexaplex vaccine provides significant advantages in the context of modern vaccines, commercial Flublok and Fluad were obtained to perform direct comparator vaccines. First, the reactivity of antigens bound to liposomes was confirmed using monoclonal antibodies specific to the compositional antigens in a slot-blot assay. Detection of marked antibodies showed significant binding to each antigen on hexaplex liposomes. Similar detection levels were observed in commercial Fluad vaccines, and Flublok vaccines showed results consistent with the HA-only composition of the vaccine (Figures 4B and S3). For reasons that were not identified, Fluad showed lower reactivity with N1 and N2 antibodies than the hexaplex; this may be due to a greater than expected mismatch between egg-derived and recombinant NA which resulted in lower monoclonal antibody binding, or possible actual differences in NA content. Based on ELISA antibody titers following vaccination, antibodies produced in response to Fluad were still effective at binding to matched NA. Hexaplex vaccines induced greater antibody titers relative to contemporary Flublok (recombinant) and Fluad (adjuvanted inactivated) vaccines (Figures 4C and S2). Hemagglutinin inhibition by serum from vaccinated mice was comparable between vaccines, with hexaplex yielding significantly greater HAI against the test H3 and B Victoria strains, and Flublok and Fluad performing better against the test H1 and B Yamagata strains. Of note, the hexaplex vaccine yielded relatively high inhibition against B Yamagata despite not containing a B Yamagata antigen, representing a quadrivalent inhibition profile despite possessing only trivalent antigen formulation (Figure 4D). Furthermore, hexaplex

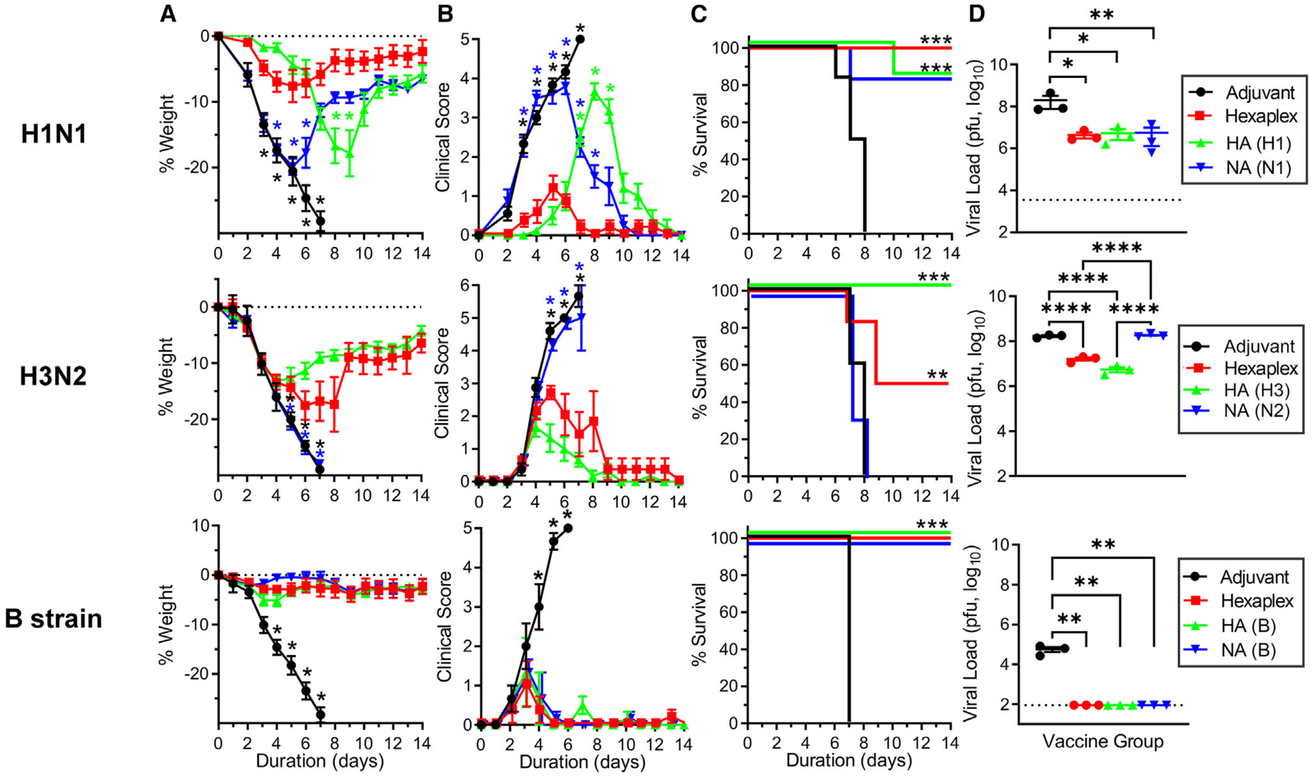

Figure 3. HA, NA, and hexaplex liposome vaccines are protective against viral challenge in mice

Viral challenge against mouse-adapted A/California/04/2009 H1N1 (top), mouse-adapted A/Hong Kong/1/1968 H3N2 (middle), and mouse-adapted B/Malaysia/2506/2004 (bottom) with hexaplex particle vaccines, 1.8 μg total antigen. BALB/c mice were vaccinated on days 0 and 21 and challenged on day 42. Measurements for body weight loss from the day of viral challenge (A), clinical score assessment (B), survival above 25% weight loss threshold (C), and lung viral load in mice sacrificed on day 4 post challenge (D) were recorded. Dotted lines in (D) indicate lower limit of detection. Statistical analysis for (A), (B), and (D) was performed by one-way ANOVA with Tukey's post hoc multiple comparisons. Statistical analysis for (C) was performed by log-rank test. Asterisks on survival curves indicate significance relative to control curves. Viral load analysis was performed on log-transformed data. Asterisks indicate *$p < 0.05$, **$p < 0.01$, ***$p < 0.005$, and ****$p < 0.001$. Color-matched asterisks compare corresponding colored data point to the hexaplex data at that point in time. Biological replicates of n = 3 for viral load assays and n = 6 for survival challenges were used.

achieved significantly higher antigen-specific splenocyte activity than vaccination with either Flublok or Fluad, which we suspect is due to the inclusion of lipid adjuvant QS21 (Figure 4E). Additional serological analysis was performed on another cohort of BALB/c mice against a panel of heterologous seasonal influenza strains, namely A/California/04/2009 (H1N1), A/New York/39/2012 (H3N2), and B/Washington/02/2019 (Victoria lineage) viruses. These results indicated that in heterologous strain comparisons, hexaplex vaccine again achieved significantly higher IgG titers; more interestingly, H1N1 HAI titer remained stable in hexaplex whereas they were diminished in Flublok and Fluad groups, and inhibitory assay results showed hexaplex achieved significant advantage for H3N2 in HAI and all strains in NAI when compared to Fluad and especially Flublok (Figure S4). NAI titers for hexaplex significantly exceeded those of Flublok, likely corresponding to the effect of steric hindrance from HA antibodies alone, since Flublok lacks NA.

Vaccine efficacy compared to contemporary vaccines

The hexaplex vaccine was compared directly to Flublok and Fluad in viral challenges against mouse-adapted H1N1 (10× LD_{50}) and mouse-adapted H3N2 (2.5× LD_{50}). Fluad was diluted to provide relative HA antigen doses consistent with the HA dosage delivered by the hexaplex vaccine (0.3 μg per HA). Flublok was diluted to use a 3-fold higher HA dose (0.9 μg per HA), which is consistent with the scale factor between Fluad and Flublok in commercial vaccines. Liposomal vaccines were effective at reducing maximum body weight loss against both H1N1 and H3N2, contrasted against Fluad, which only provided effective body weight protection against H1N1, and Flublok, which did not provide significant protection in either challenge (Figure 5A). Clinical scoring of mice demonstrated that the hexaplex vaccine was able to reduce symptoms against H1N1, while disease courses for H3N2-infected mice were not significantly distinct (Figure 5B). Mice were sacrificed upon reaching a 25% reduction from initial body weight. Based on this criterion, only hexaplex and Fluad prevented complete mortality against H1N1, and against H3N2 only hexaplex vaccine yielded survival (Figure 5C). Lung viral load in mice sacrificed on day 4 post infection showed a slight reduction in viral particles in the lungs of hexaplex- and Fluad-vaccinated mice Figure 5D). Additionally, preliminary studies with the hexaplex vaccine were performed

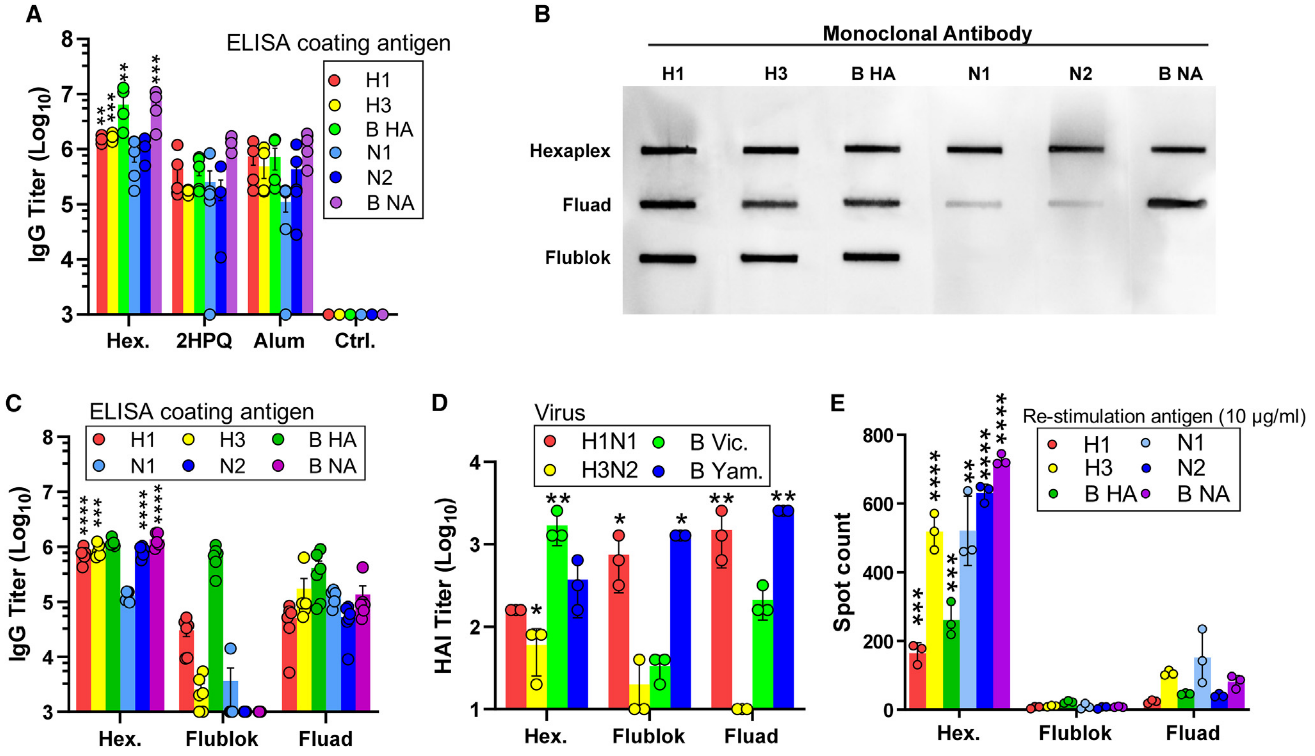

Figure 4. Hexaplex composition and immunogenicity compared with contemporary vaccines

Serum was analyzed from mice vaccinated on day 0 and day 21 with 50-μL doses containing 0.3 μg of each antigen in the hexaplex vaccine, Fluad diluted to 0.3 μg of each HA, or Flublok diluted to 0.9 μg of each HA.

(A) Multiple admixtures of all antigens with binding, non-binding (2HPQ) liposomes, and alum adjuvant-induced binding antibodies in mice.

(B) Monoclonal antibodies were able to detect all six antigens in functional conformation in hexaplex, Fluad vaccine (with notable reduction in N1 and N2 binding), and the three HA antigens contained in the Flublok vaccine.

(C) Hexaplex liposomes induced greater immune responsiveness in splenocyte cells than Flublok or Fluad.

(D) Hemagglutination inhibition differed between formulations, with hexaplex vaccine favoring higher H3 and B Victoria inhibition, while Fluad and Flublok elicited higher H1 and B Yamagata inhibition.

(E) Splenocyte activation in response to antigen stimulation was heightened in hexaplex vaccine, owing to QS21 adjuvant incorporation.

Statistical analysis was performed by two-way ANOVA with multiple comparisons. Asterisks indicate significance relative to hexaplex; asterisks over hexaplex bars indicate significant increase over all other groups, while asterisks over comparator bars indicate significant increase over hexaplex. Asterisks indicate *$p < 0.05$, **$p < 0.01$, ***$p < 0.005$, and ****$p < 0.001$. Biological replicates of $n = 5$ were used.

in ferrets, a preferred model organism for influenza virus study. Favorable serological data were observed as vaccination with hexaplex liposomes elicited greater antibody titers against five of the six antigens than either Flublok or Fluad by a significant margin. In HAI, mean inhibition titer was highest among the hexaplex vaccination groups, and in NAI, inhibition titers were similar between hexaplex and Fluad (Figure S5).

Protection conferred by serum transfer

To assess whether effective protection was provided by antibody responses to the hexaplex vaccine, serum from vaccinated mice was transferred to naive mice prior to challenging with a 10× LD_{50} inoculum of mouse-adapted A/California/04/2009 H1N1 virus. At 42 days post primary vaccination with a boost on day 21, a cohort of eight mice in four groups corresponding to hexaplex, HA, NA, and Fluad vaccines were exsanguinated, and serum was pooled to generate the donor serum stock, which was administered 2 h prior to viral challenge by intraperitoneal injection to nine mice in four groups. For each group, three mice were sacrificed on day 4 for lung tissue analysis, and the remaining six mice per group were observed for the complete 14-day infection time course. Although the HA-only liposomes were the only ones to yield a marginal increase in body weight retention over the other experimental vaccines during the challenge, both hexaplex and HA liposomes enhanced the immune response to a degree whereby recovery was possible in surviving animals (Figure 6A). Despite achieving high clinical scores among experimental groups, following day 9 the animals treated with serum from HA-vaccinated mice underwent a dramatic recovery phase, with surviving hexaplex-serum-treated animals achieving a recovery in parallel with their body weight recovery relative to HA-treated mice (Figure 6B). Of the six mice that underwent the full 14-day challenge, four HA-serum-treated mice and three hexaplex-serum-treated mice survived based on the 25% body weight loss criteria, while all control, NA-serum-treated, and Fluad-serum-treated mice fell below the threshold

CellPress
OPEN ACCESS

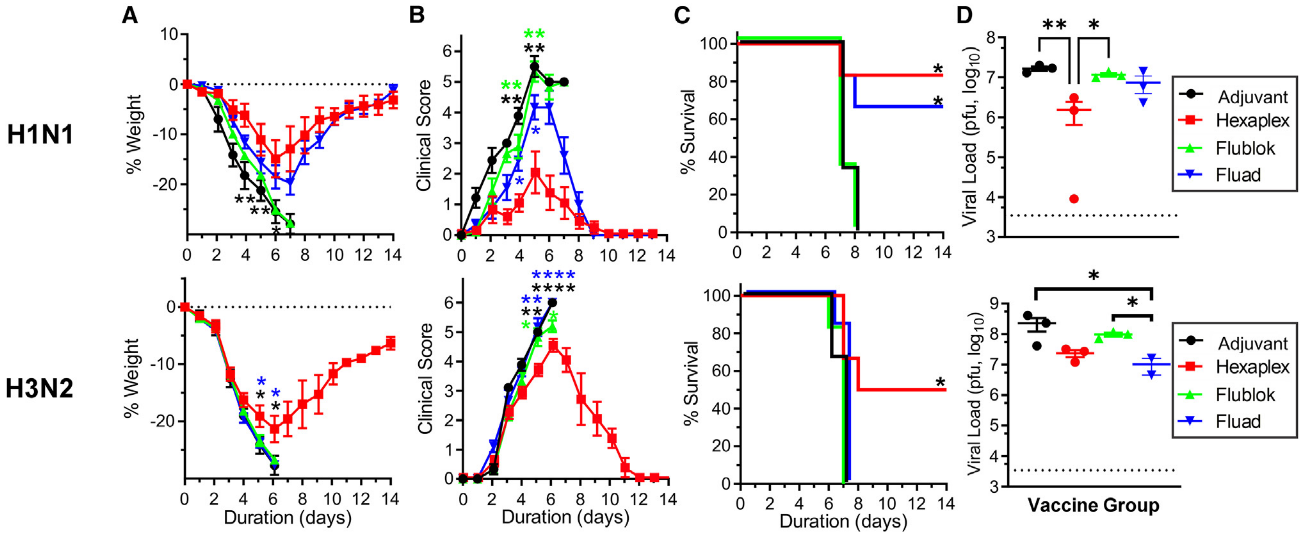

Figure 5. Comparison of contemporary vaccines against viral challenge in mice

Viral challenge against mouse-adapted A/California/04/2009 H1N1 (top) and A/Hong Kong/1/1968 H3N2 (bottom) comparing hexaplex nanoparticle vaccine against available Flublok and Fluad formulations. Mice were vaccinated on days 0 and 21 with 0.3 μg of each antigen in hexaplex (a total of 1.8 μg of antigen), Fluad diluted to 0.3 μg of each HA, or Flublok diluted to 0.9 μg of each HA. Hexaplex achieved marginally higher body weight protection (A), reduced clinical scores (B), increased survival (C), and greater overall survival (D) of animals than Flublok or Fluad. Dotted lines in (D) indicate lower detection limit. Statistical analysis for (A), (B), and (D) was performed by one-way ANOVA with Tukey's post hoc multiple comparisons. Statistical analysis for (C) was performed by log-rank test. Viral load analysis was performed on log-transformed data. Asterisks indicate *$p < 0.05$, **$p < 0.01$, ***$p < 0.005$, and ****$p < 0.001$. Color-matched asterisks compare corresponding colored data point to the hexaplex data at that time. Survival curve asterisks indicate significance relative to control curve. Biological replicates of n = 3 for viral load assays and n = 6 for survival challenges were used.

for survival (Figure 6C). Under these conditions, no significant difference was observed in the viral load in the lungs at 4 days post infection (Figure S6).

DISCUSSION

The rapid rate of evolution among influenza strains necessitates a continuous effort to improve the breadth and efficacy of influenza vaccines. The development of recombinant antigen vaccines has been a relatively recent advancement, with potential for precise vaccines that overcome limitations of traditional virus-derived vaccines. There has been recent interest in the form of nanoparticulate antigen carriers and immunostimulants. Building upon prior research, antigen-bearing liposomes have yielded promising results in the protective efficacy elicited by the adjuvanted vaccine.

In serological analysis, adjuvanted liposomes are found to elicit cross-reactive antibodies between HA candidate proteins when coupled with only a single HA serotype, with similar cross-reactivity reflected in NA groups. Multimerization domains are observed to be immunogenic, potentially confounding ELISA binding titer analyses.[25] Although this cross-reactivity decreased with more divergent strains, levels of cross-reactivity were observable in antibodies elicited by H3, N1, and N2 antigens using ELISA coating antigens lacking multimerization domains and at a level higher than Flublok or Fluad (Figure S2). Furthermore, cross-reactive HAI was observed in Figure 2B between H1 and H3 when utilizing the A/Darwin recombinant antigen as well as between the two influenza B lineages when utilizing B/Austria antigen. As hexaplex studies expand, we hope that these results will be borne out by future serological assays, as it would represent an interesting prospect for potential heterosubtypic protection resulting from the vaccine design. Future assay design may also investigate polymeric recombinant antigens using alternative multimerization domains from sequentially distinct membrane proteins as a means of preventing the detection of multimerization domain antibodies while preserving multimeric epitopes.

Hexaplex liposomes provide an adjuvant effect that results in effective protection against each representative seasonal strain of influenza in challenge, whereas unadjuvanted recombinant antigens do not. Furthermore, hexaplex provides comparable or superior outcomes to comparator vaccines in virus challenge, with the exception of A/Hong Kong H3N2, where hexaplex provides lower protection than H3 alone yet significantly increased survival over the control. Of note, mouse-adapted A/Hong Kong challenge virus possesses the lowest protein sequence homology among homosubtypic HA proteins (Table S1), representing a highly divergent antigen mismatch scenario. In this case, hexaplex vaccine performed favorably compared to Flublok and Fluad vaccines, which are unable to provide protection against this viral strain. Serological studies indicate further potential, as high antibody titers capable of binding viruses within the same serotype across disparate seasons were observed in multiple ELISA assays against heterologous seasonal influenza strains and even achieved greater inhibitory titers in HAI and NAI assays (Figures S2 and S4). These results seem to validate the potential of this platform for usage as a broadly protective vaccine for multiple seasonal strains.

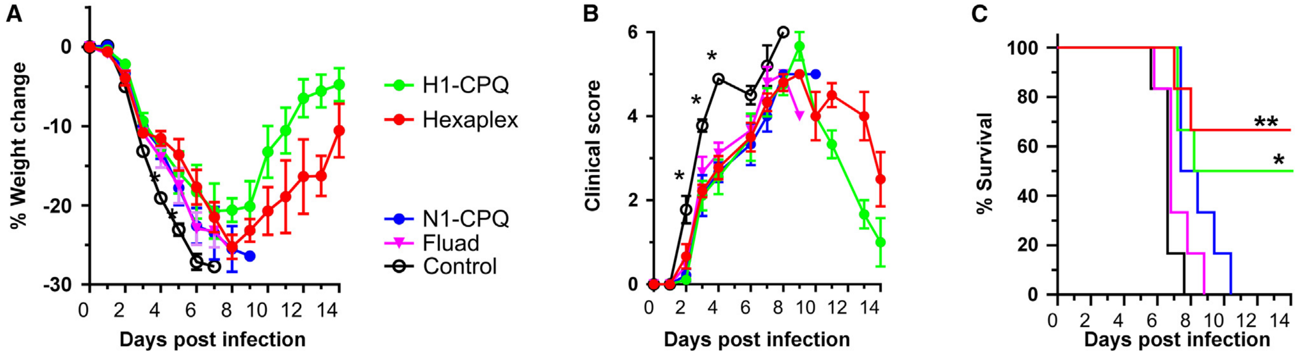

Figure 6. Serum transfer protects mice from virus challenge

Mice were vaccinated as in previous challenges, after which vaccinated mice were sacrificed, serum was collected, pooled, and 300 μL was introduced into naive mice intraperitoneally 2 h prior to challenge with A/California/04/2009 H1N1. Mice experienced similar weight loss (A) and clinical score time courses (B), but only mice transferred serum from hexaplex or H1 liposome vaccinated animals resulted in significant increases in survival (C). Statistical analysis for (A) and (B) was performed by one-way ANOVA with Tukey's post hoc multiple comparisons. Statistical analysis for (C) was performed by log-rank test. Asterisks indicate *$p < 0.05$ and **$p < 0.01$. Asterisks in (B) show comparison of the control group to the hexaplex group. Survival curve asterisks indicate significance relative to control curve. Biological replicates of n = 9 were used.

There are still a number of open questions following this research. While each antigen comprised an equal part in the ratio of total antigens applied to liposomes, serological analysis revealed an imbalance in relative response between antigen strains. H1, H3, and N2 all possessed a decreased IgG titer in hexaplex when compared to their single-antigen formulations, and HAI and NAI levels were similarly decreased. While the hexaplex vaccine showed superior efficacy in direct challenge, the antibody-based protection was revealed to be inferior to HA-only vaccine. Similar protective inefficiency was also observed when mice were challenged with H3N2 virus. While the reason for these observations is not known definitively, a contributing factor is likely that the hexaplex vaccines are designed to possess the same total antigenic dose as the single-antigen vaccines and, thus, only one-sixth of the quantity could be presented compared to its single-antigen counterpart. It may be possible to optimize the antigen dosage to maximize the antibody saturation against each component in order to ensure the strongest possible response to each seasonal influenza strain. This will require further investigation, including potential dose-sparing challenge to ascertain the ideal antigen dosage for broad trivalent protection.

The hexaplex formulation investigated contains HA and NA against three seasonal influenza strains, despite the fact that modern vaccines against influenza have been predominantly quadrivalent. However, during the worldwide pandemic of SARS-CoV-2 there was a profound decline in the circulation of B Yamagata lineage influenza, leading to speculation that the B Yamagata lineage has become extinct.[26] In the most recent 2023–2024 WHO influenza vaccine recommendations, the B Yamagata strain recommendation has been dropped. Thus, it seems plausible that seasonal influenza vaccines are likely to reconverge on a trivalent future. Furthermore, if B Yamagata should re-emerge, the hexaplex formulation with only a B Victoria representative induced antibodies reactive and neutralizing to both B Victoria and B Yamagata (Figures 2B and 2C). We had predicted this outcome based on the approximate 92% sequence homology between HA and 94% homology between NA of the B influenza, compared with the homology of strains of H1N1 and H3N2 for which the hexaplex antigens successfully induced neutralization (Table S1). Therefore, it is possible that a hypothetical re-emergence of B Yamagata may be controlled by vaccines inducing broadly reactive B influenza antibodies rather than reformulating to include specific components to induce response against B Yamagata lineage.

The interactions between antigens in a single-particle vaccine formulation have yet to be fully understood; however, it is encouraging that a nanoparticle with conventional refrigeration storage, capable of rapidly forming biostable bonds with a variety of His-tagged antigens under simple incubation conditions, performed favorably in lethal mouse challenge against influenza compared to existing human-approved influenza in a head-to-head comparison at equivalent HA doses. The relative contribution of PHAD adjuvant, QS21 adjuvant, particle size, liposome display, NA inclusion, and antigen density as well as each individual antigen was not assessed here but is an area of interest. When combined with rapid-output methods for the production of recombinant antigens, such as the baculovirus translation methods currently employed for the production of soluble antigens for Flublok vaccine production,[27,28] hexaplex liposomes could provide a large-scale manufacturing pathway for clinical applications. The successful incorporation of lipid-based adjuvants indicates the potential to elicit a more pronounced immune response, with applications possibly extending to the most vulnerable populations such as the elderly and immunodeficient. Furthermore, the full capacity of multiplexed liposomes has yet to be investigated. In a previous study, it was reported that a multiplex candidate vaccine possessing ten antigens could achieve a balanced and measurable response against each antigen[18]; therefore, it is possible that multiplexed liposomal design could be expanded beyond the scope of a single season. As previously stated, results within this study seem to indicate the presence of cross-reactive antibodies; we hope to tailor the liposomal vaccine to reliably elicit these antibodies and determine

the full breadth of protection that could be yielded across multiple seasons. Ideally, interrogation of the potential of this liposomal platform could lead to vaccines with both seasonal and pandemic candidate protection and, with further study, perhaps yield a breakthrough in the field of broadly protective, "universal" influenza vaccines.

Limitations of the study

While the studies presented herein elucidate significant potential for antigen-binding liposomal vaccines, there are several factors that have imposed limitations on these studies. Currently, the experimental vaccines used were formulated in the absence of a method for quantitatively measuring the antigen quantities bound to liposomes following incubation. Such methods must be developed to achieve approved applications for this adjuvant system. This could be facilitated by the use of a panel of antigen-specific and non-cross-reactive monoclonal antibodies such as those utilized for qualitative assessment of antigen binding by slot-blot detection, as in Figures 1D and 4B. Quantification of liposome-bound fractions of antigens might further be facilitated via microcentrifugal filtration assays to separate soluble from liposome-bound antigen. Splenocyte activation as an indicator of cellular immunity was investigated within this study; however, additional components of cell-mediated immunity have yet to be thoroughly explored. Future work will involve interrogations of T cell protective efficacy as well as the contributions of antibody-dependent cell killing of infected cells.[29,30] Additionally, while the lungs of infected animals were assessed only on day 4, future studies should also assess the viral lung titer around day 7, as this is the point that corresponds with peak infection and would provide a more complete assessment of viral kinetics.

The scope of this study was largely limited to immunogenicity and challenge studies in young mice with mouse-adapted strains of influenza, and preliminary immunogenicity assessment in ferrets. This emphasis on murine studies limits the number and variety of influenza viruses that can be applied and tested in challenges. Due to the susceptibility of ferrets to influenza virus, ferret challenge studies should be undertaken in preclinical analysis to draw any conclusions about the efficacy of this liposomal vaccine platform with more human-relevant virus strains. Syrian hamsters are another animal model of interest, especially for the investigation of H3N2 viruses in challenge, as the development of mouse-adapted H3N2 viruses faces significant challenges.[31] The evaluation of the vaccine would further benefit from testing in non-human primates to determine vaccine performance in a model organism whose immune response better reflects that of humans. Translation to human testing poses other questions. The manufacture of a multivalent liposomal nanoparticle will be costly and will require significant development of manufacturing and characterization techniques, especially as vaccine antigens should be remade and reformulated on an annual basis. Ideally, these methods will prove favorable relative to the cost of traditional influenza vaccine production. Furthermore, while laboratory animals are generally immunologically naive, human pre-exposure to influenza viruses is certain to introduce bias to the immune response elicited by vaccines which could be difficult to predict. Despite these limitations, the hexaplex vaccine presented shows a remarkable capacity to provide protective immunity against representatives of each major seasonal influenza virus, H1N1, H3N2, and B Victoria influenza, at doses for which commercially available Flublok and Fluad vaccines were revealed to be relatively less effective in mice, making it promising for further testing and development.

Conclusions

Hexaplex liposomes bearing three HAs and three NA multimeric protein antigens were successful at eliciting protection against representative mouse-adapted influenza virus strains for H1N1, H3N2, and B Victoria lineages. Even at one-sixth the strain-specific antigen dose, hexaplex liposomes yielded superior protective efficacy against both H1 and N1 alone and fell within protective margins of the single-antigen H3, B HA, and B NA vaccinations against subtype-matched viruses. When compared to dose-normalized Flublok and Fluad vaccines, hexaplex liposomes provided superior protection and survival in both direct vaccination and serum-transfer scenarios. While numerous nanoparticle vaccines against influenza have been proposed to date, this methodology includes the advantages of rapid integration and co-display of HA and NA antigens as well as the co-delivery of lipid adjuvants including PHAD and QS21. A liposomal approach also might reduce the chance for off-target antibody production against structural proteins, as in protein-cage nanoparticles. In other words, the use of a protein-free lipid scaffold reduces the likelihood of developing scaffold-specific immune responses with repeated immunization. The production efficiency of hexaplex combined with the capacity to elicit cross-reactive and cross-inhibitory antibodies when combined with influenza antigens suggests that antigen-binding liposomes hold potential in clinical applications and possible future avenues for broadly protective vaccine designs.

STAR★METHODS

Detailed methods are provided in the online version of this paper and include the following:

- KEY RESOURCES TABLE
- RESOURCE AVAILABILITY
  - Lead contact
  - Materials availability
  - Data and code availability
- EXPERIMENTAL MODEL AND STUDY PARTICIPANT DETAILS
  - Cell culture
  - Mouse immunizations
  - Ferret immunization
- METHOD DETAILS
  - Vaccine antigen selection
  - Liposome production
  - Vaccine preparation
  - Liposome characterization
  - Cryo-transmission electron microscopy characterization
  - Slot blot
  - Serological antibody ELISA
  - Serological antibody inhibition and neutralization

- ○ ELISPOT
- ○ Influenza virus plaque assay in MDCK cells
- ● QUANTIFICATION AND STATISTICAL ANALYSIS

SUPPLEMENTAL INFORMATION

Supplemental information can be found online at https://doi.org/10.1016/j.xcrm.2024.101433.

ACKNOWLEDGMENTS

We thank Dr. James Stevens of the Centers for Disease Control and Prevention for valuable discussion on antigen design and Martine Boyer for providing antigens. We thank Dr. James Kobie for anti-NA antibodies. We acknowledge and thank staff members of the Parrish Facility for Electron Microscopy Research (FEMR) at McGill University for helping in microscope operation and cryo-TEM image data collection. FEMR is supported by the Canadian Foundation for Innovation, the Quebec government, and McGill University. We also thank Dr. Richard J. Webby of Saint Jude Children's Research Hospital (Memphis, TN) for supplying the mouse-adapted influenza A/California/04/2009pdm (H1N1) and Dr. Florian Krammer of the Icahn School of Medicine at Mount Sinai for supplying mouse-adapted B/Malaysia/1359417/2021 (Victoria lineage). This study was funded, in part, by NIH grants 1R41 AI149954 and 5R01 HL151498. This material is also the result of work supported with resources and the use of facilities at the Veterans Administration Western New York Healthcare System, Buffalo, NY. The contents of this manuscript do not represent the views of the Department of Veterans Affairs or the United States Government. Experimental protocols involving mice, including immunizations and subsequent challenge studies, were reviewed and approved by the University at Buffalo and the VAWNYHS.

AUTHOR CONTRIBUTIONS

Conceptualization, Z.R.S. and J.F.L.; methodology, Z.R.S., B.A.D., and J.F.L.; investigation, Z.R.S., J.R., W.-C.H., Y.S., S.Z., Y.L., Q.L., D.A., H.L.K., J.O., and B.A.D.; project administration. J.F.L.; supervision, B.A.D.; resources, B.A.D. and J.F.L.; writing – original draft, Z.R.S.; writing – review & editing, Z.R.S., B.A.D., and J.F.L.

DECLARATION OF INTERESTS

J.F.L. and W.-C.H. hold competing interests in POP Biotechnologies. W.-C.H. and H.L.K. are employed by POP Biotechnologies. Patents related to this work have been filed by the State University of New York.

Received: July 3, 2023
Revised: November 29, 2023
Accepted: January 25, 2024
Published: February 23, 2024

REFERENCES

1. Centers for Disease Control and Prevention (2023). 2022-2023 U.S. Flu Season: Preliminary In-Season Burden Estimates. https://www.cdc.gov/flu/about/burden/preliminary-in-season-estimates.htm.
2. Partridge, J., and Kieny, M.P. (2013). Global production capacity of seasonal influenza vaccine in 2011. Vaccine *31*, 728–731. https://doi.org/10.1016/j.vaccine.2012.10.111.
3. Gasparini, R., Amicizia, D., Lai, P.L., Rossi, S., and Panatto, D. (2013). Effectiveness of adjuvanted seasonal influenza vaccines (Inflexal V ® and Fluad ®) in preventing hospitalization for influenza and pneumonia in the elderly: a matched case-control study. Hum. Vaccin. Immunother. *9*, 144–152. https://doi.org/10.4161/hv.22231.
4. Pérez Rubio, A., and Eiros, J.M. (2018). Cell culture-derived flu vaccine: Present and future. Hum. Vaccin. Immunother. *14*, 1874–1882.
5. Wong, S.-S., and Webby, R.J. (2013). Traditional and new influenza vaccines. Clin. Microbiol. Rev. *26*, 476–492.
6. Douglas, C., Powers, M.C.M., Hanscome, P.J., and Freda Pietrobon, P.J. (1995). Cytotoxic T Lymphocyte Responses to a Liposome-Adjuvanted Influenza A Virus Vaccine in the Elderly. J. Infect. Dis. *172*, 5.
7. Sia, Z.R., Miller, M.S., and Lovell, J.F. (2021). Engineered Nanoparticle Applications for Recombinant Influenza Vaccines. Mol. Pharm. *18*, 576–592.
8. Trombetta, C.M., Marchi, S., and Montomoli, E. (2022). The baculovirus expression vector system: a modern technology for the future of influenza vaccine manufacturing. Expert Rev. Vaccines *21*, 1233–1242. https://doi.org/10.1080/14760584.2022.2085565.
9. Chen, Y.-Q., Wohlbold, T.J., Zheng, N.-Y., Huang, M., Huang, Y., Neu, K.E., Lee, J., Wan, H., Rojas, K.T., Kirkpatrick, E., et al. (2018). Influenza infection in humans induces broadly cross-reactive and protective neuraminidase-reactive antibodies. Cell *173*, 417–429.e10.
10. Doyle, T.M., Hashem, A.M., Li, C., Van Domselaar, G., Larocque, L., Wang, J., Smith, D., Cyr, T., Farnsworth, A., He, R., et al. (2013). Universal anti-neuraminidase antibody inhibiting all influenza A subtypes. Antiviral Res. *100*, 567–574.
11. Kim, K.-H., Lee, Y.-T., Park, S., Jung, Y.-J., Lee, Y., Ko, E.-J., Kim, Y.-J., Li, X., and Kang, S.-M. (2019). Neuraminidase expressing virus-like particle vaccine provides effective cross protection against influenza virus. Virology *535*, 179–188. https://doi.org/10.1016/j.virol.2019.07.008.
12. Stadlbauer, D., Zhu, X., McMahon, M., Turner, J.S., Wohlbold, T.J., Schmitz, A.J., Strohmeier, S., Yu, W., Nachbagauer, R., Mudd, P.A., et al. (2019). Broadly protective human antibodies that target the active site of influenza virus neuraminidase. Science *366*, 499–504.
13. Johansson, B.E. (1999). Immunization with influenza A virus hemagglutinin and neuraminidase produced in recombinant baculovirus results in a balanced and broadened immune response superior to conventional vaccine. Vaccine *17*, 2073–2080. https://doi.org/10.1016/S0264-410X(98)00413-7.
14. Kosik, I., and Yewdell, J.W. (2019). Influenza hemagglutinin and neuraminidase: Yin–Yang proteins coevolving to thwart immunity. Viruses *11*, 346.
15. Kanekiyo, M., Wei, C.-J., Yassine, H.M., McTamney, P.M., Boyington, J.C., Whittle, J.R.R., Rao, S.S., Kong, W.-P., Wang, L., and Nabel, G.J. (2013). Self-assembling influenza nanoparticle vaccines elicit broadly neutralizing H1N1 antibodies. Nature *499*, 102–106. https://doi.org/10.1038/nature12202.
16. Huang, W.C., Deng, B., Lin, C., Carter, K.A., Geng, J., Razi, A., He, X., Chitgupi, U., Federizon, J., Sun, B., et al. (2018). A malaria vaccine adjuvant based on recombinant antigen binding to liposomes. Nat. Nanotechnol. *13*, 1174–1181. https://doi.org/10.1038/s41565-018-0271-3.
17. Shao, S., Geng, J., Ah Yi, H., Gogia, S., Neelamegham, S., Jacobs, A., and Lovell, J.F. (2015). Functionalization of cobalt porphyrin-phospholipid bilayers with his-tagged ligands and antigens. Nat. Chem. *7*, 438–446. https://doi.org/10.1038/nchem.2236.
18. Sia, Z.R., He, X., Zhang, A., Ang, J.C., Shao, S., Seffouh, A., Huang, W.-C., D'Agostino, M.R., Teimouri Dereshgi, A., Suryaprakash, S., et al. (2021). A liposome-displayed hemagglutinin vaccine platform protects mice and ferrets from heterologous influenza virus challenge. Proc. Natl. Acad. Sci. USA *118*. e2025759118.
19. Sia, Z.R., Chiem, K., Huang, W.-C., Seffouh, A., Teimouri Dereshgi, A., Hogan, T., Ortega, J., Davidson, B.A., Martinez-Sobrido, L., and Lovell, J.F. (2022). Respiratory Vaccination with Hemagglutinin Nanoliposomes Protects Mice from Homologous and Heterologous Strains of Influenza Virus. J. Virol. *96*, e0100622–e0101022.
20. Wang, N., Chen, M., and Wang, T. (2019). Liposomes used as a vaccine adjuvant-delivery system: From basics to clinical immunization. J. Control. Release *303*, 130–150. https://doi.org/10.1016/j.jconrel.2019.04.025.
21. Lovell, J.F., Baik, Y.O., Choi, S.K., Lee, C., Lee, J.-Y., Miura, K., Huang, W.-C., Park, Y.-S., Woo, S.-J., Seo, S.H., et al. (2022). Interim analysis

CellPress
OPEN ACCESS

from a phase 2 randomized trial of EuCorVac-19: a recombinant protein SARS-CoV-2 RBD nanoliposome vaccine. BMC Med. *20*, 462. https://doi.org/10.1186/s12916-022-02661-1.

22. Lovell, J.F., Miura, K., Baik, Y.O., Lee, C., Lee, J.Y., Park, Y.S., Hong, I., Lee, J.H., Kim, T., Seo, S.H., et al. (2024). One-year antibody durability induced by EuCorVac-19, a liposome-displayed COVID-19 receptor binding domain subunit vaccine, in healthy Korean subjects. Int. J. Infect. Dis. *138*, 73–80. https://doi.org/10.1016/j.ijid.2023.11.004.

23. World Health Organization (2022). Recommended Composition of Influenza Virus Vaccines for Use in the 2022-2023 Northern Hemisphere Influenza Season.

24. Chen, Y.-Q., Lan, L.Y.-L., Huang, M., Henry, C., and Wilson, P.C. (2019). Hemagglutinin Stalk-Reactive Antibodies Interfere with Influenza Virus Neuraminidase Activity by Steric Hindrance. J. Virol. *93*, e01526–18. https://doi.org/10.1128/jvi.01526-01518.

25. Sliepen, K., van Montfort, T., Melchers, M., Isik, G., and Sanders, R.W. (2015). Immunosilencing a highly immunogenic protein trimerization domain. J. Biol. Chem. *290*, 7436–7442. https://doi.org/10.1074/jbc.M114.620534.

26. Koutsakos, M., Wheatley, A.K., Laurie, K., Kent, S.J., and Rockman, S. (2021). Influenza lineage extinction during the COVID-19 pandemic? Nat. Rev. Microbiol. *19*, 741–742. https://doi.org/10.1038/s41579-021-00642-4.

27. Cox, M.M.J., and Hashimoto, Y. (2011). A fast track influenza virus vaccine produced in insect cells. J. Invertebr. Pathol. *107*, S31–S41. https://doi.org/10.1016/j.jip.2011.05.003.

28. Cox, M.M.J., Izikson, R., Post, P., and Dunkle, L. (2015). Safety, efficacy, and immunogenicity of Flublok in the prevention of seasonal influenza in adults. Ther. Adv. Vac. *3*, 97–108.

29. Brown, D.M., Dilzer, A.M., Meents, D.L., and Swain, S.L. (2006). CD4 T cell-mediated protection from lethal influenza: perforin and antibody-mediated mechanisms give a one-two punch. J. Immunol. *177*, 2888–2898.

30. Von Holle, T.A., and Moody, M.A. (2019). Influenza and antibody-dependent cellular cytotoxicity. Front. Immunol. *10*, 1457.

31. Iwatsuki-Horimoto, K., Nakajima, N., Ichiko, Y., Sakai-Tagawa, Y., Noda, T., Hasegawa, H., and Kawaoka, Y. (2018). Syrian Hamster as an Animal Model for the Study of Human Influenza Virus Infection. J. Virol. *92*, e01693-17. https://doi.org/10.1128/jvi.01693-01617.

STAR★METHODS

KEY RESOURCES TABLE

REAGENT or RESOURCE	SOURCE	IDENTIFIER
Antibodies		
Anti-Influenza A Nuceloprotein HT-103	Kerafast	EMS010
Goat Anti-Mouse HRP	GenScript	A00160
Goat Anti-Ferret HRP	Novus Biologics	nb7224
Anti-H1 A/Brisbane/59/2007	International Reagent Resource	FR-494
Anti-H3 A/Victoria/361/2011	International Reagent Resource	FR-1122
Anti-B HA B/Brisbane/60/2008	International Reagent Resource	FR-1337
Anti-N1 A/Brisbane/59/2007	International Reagent Resource	FR-941
Anti-N2 A/Perth/16/2009	International Reagent Resource	FR-1156
Anti-B NA hmAb	Dr. James Kobie	1092E10
Chemicals		
Arachis hypogaea Lectin (PNA) – peroxidase conjugate (HRP)	Sigma Aldritch	L7759
Bovine Serum Albumin (BSA)	Sigma-Aldrich	9048-46-8
Cobalt porphyrin phospholipid (CoPoP)	POP Biotechnologies	N/A
Dulbecco's modified Eagle's media (DMEM)	Corning	10-013-CM
Fetal Bovine Serum (FBS)	Corning	35-010-CF
Fetuin	Sigma-Aldrich	9014-81-7
Penicillin-Streptomycin-Glutamine (PSG)	Thermo Fisher Scientific	10378016
Phosphate Buffered Saline (PBS)	Thermo Fisher Scientific	10010023
1,2-dioleoyl-*sn*-glycero-3-phosphocholine (DOPC)	Corden Pharma	LP-R4-070
PhytoChol	Wilshire Technologies	N/A
Saponin QS21	Desert King	N/A
Monophosphoryl Hexa-acyl Lipid A, 3-Deacyl	Avanti	699855P
Aluminum Hydroxide Hydrogel	VWR	21645-51-2
Receptor destroying enzyme (RDE II)	Hardy Diagnostics	370013
TMB One Component Microwell Substrate	SouthernBiotech	0410–01
Tween 20	Sigma Aldritch	P1379
VisiGlo HRP Substrate	VWR	97063–148
Commercial assay kits		
ELISpot Assay Kit	ImmunoSpot	https://immunospot.com/mouse-ifn-gamma-single-color-elispot.html
Experimental models		
Madin-Darby canine kidney (MDCK) cells	American Type Culture Collection (ATCC)	CCL-34
CD-1 outbred mice	Envigo	030
BALB/c inbred white mice	Charles River	555
Fitch Ferrets	Bioqual (Rockville, MD)	N/A
Software		
GraphPad Prism 9	GraphPad	https://www.graphpad.com/
SerialEM	University at Colorado Boulder	https://bio3d.colorado.edu/SerialEM/
Recombinant proteins		
A/Victoria/2570/2019 (H1N1) - HA & NA antigens	Nexelis Q2 Solutions	https://nexelis.com/
A/Darwin/6/2021 (H3N2) - HA & NA antigens	Nexelis Q2 Solutions	https://nexelis.com/
B/Austria/1359417/2021 (Victoria) - HA & NA antigens	Nexelis Q2 Solutions	https://nexelis.com/

(*Continued on next page*)

CellPress
OPEN ACCESS

Continued

REAGENT or RESOURCE	SOURCE	IDENTIFIER
HA1 from A/Wisconsin/588/2019 (H1N1)	CDC	N/A
HA from A/Kansas/14/2017 (H3N2)	eEnzyme	IA-H3-K17P
HA1 from B/Brisbane/60/2008 (Victoria)	eEnzyme	IB-HA1-608P
HA2 from B/Brisbane/60/2008 (Victoria)	eEnzyme	IB-HA2-608P
NA from A/Wisconsin/588/2019 (H1N1)	eEnzyme	IA-NA1-W19P
NA from A/Hong Kong/45/2019 (H3N2)	eEnzyme	IA-NA3-HK45P
NA from B/Colorado/06/2017	eEnzyme	IB-NA-C17P
Vaccines		
FluAd Quadrivalent	Sanofi Pasteur Inc.	N/A
FluBlok Quadrivalent	Sanofi Pasteur Inc.	N/A
Virus strains		
A/California/04/2009pdm Mouse-Adapted (H1N1)	Saint Jude Children's Research Hospital (Memphis, TN)	N/A
A/Hong Kong/1/1968-1 Mouse-Adapted 12A (H3N2)	BEI Resources, NIAID, NIH	N/A
B/Malaysia/1359417/2021 Mouse-Adapted (Victoria)	Icahn School of Medicine at Mount Sinai, New York, NY	N/A
A/New York/39/2012 (H3N2)	International Reagent Resource	FR-1307
B/Washington/02/2019 (Victoria)	International Reagent Resource	FR-1709
B/Phuket/3073/2013 (Yamagata)	International Reagent Resource	FR-1365
Other		
HisPur™ Ni-NTA Magnetic Beads	Thermo Fisher Scientific	88831
NuPAGE gel	Bio-Rad	456–8084
NuPAGE™ MES SDS Buffer Kit	Thermo Fisher Scientific	NP0060
Protein molecular weight ladder	Rockland Inc., Limerick, PA	MB-201-0200
Nitrocellulose Membrane	Thermo Fisher Scientific	77012
48-Well Slot Blot Apparatus	Bio-Rad	1706542
Cell Strainer, 70 μm mesh	VWR, North American	76327–100

RESOURCE AVAILABILITY

Lead contact

Further information and requests for resources and reagents should be directed to the lead contact, jflovell@buffalo.edu.

Materials availability

CoPoP liposomes generated in this study will be made available on request.

Data and code availability

All data reported in this paper will be shared by the lead contact upon request. This paper does not report original code. Any additional information required to reanalyze the data reported in this paper is available from the lead contact upon request.

EXPERIMENTAL MODEL AND STUDY PARTICIPANT DETAILS

Cell culture

Madin-Darby canine kidney (MDCK) cells were grown and maintained in Dulbecco's modified Eagle's media (DMEM) supplemented with 10% fetal bovine serum (FBS), 1% PSG (100 units/ml penicillin, 100 μg/mL streptomycin, 2 mM L-glutamine) at 37°C and 5% CO_2. Viral titers in the tissue culture supernatants collected at 48 h post-infection were determined by immunofocus assay (fluorescent forming units, FFU/ml) in MDCK cells. Confluent monolayers of MDCK cells (96-well plate format, 5 x 10^4 cells/well, triplicates) were infected with 10-fold dilutions of tissue culture supernatants at 33°C. Then, 12 h post-infection, the cells were washed with PBS, and individual infected cells were detected using the monoclonal antibody HT-103 against the viral nucleoprotein (NP) and were visualized using a fluorescence microscope to determine FFU/ml.

Mouse immunizations

Prior to challenge, all mice were subjected to a standard vaccination regimen involving a primary intramuscular vaccination containing 50 μL volume of mixture specified by the experiment, followed by a second booster vaccination at 21 days post-prime. Mice were challenged 42 days post-prime with an intranasal inoculation with a mouse-adapted strain of H1N1, H3N2, or B Victoria lineage influenza virus (50 μL). For H1N1 and B influenza, the inoculum dose was 2.5x or 10x LD_{50}, while for H3N2, this dose was equivalent to 2.5x LD_{50}. Prior to the reported experiment, a dose of 10x LD_{50} for H3N2 was found to result in complete mortality. Mice were monitored daily with body weight measurements and clinical score assessments. Animals that exhibited a body weight loss of 25% or greater were euthanized. Subsets of three challenge mice were sacrificed after 4 days and blood and lung tissue were collected. Briefly, following induction of anesthesia with 3% isoflurane in 100% O_2, a longitudinal incision was made, and ≈0.5 mL of blood collected from the abdominal aorta. Following 45 min of coagulation at RT the blood was centrifuged at 5,000 x g for 5 min at 4°C and the serum collected and stored at −80°C. The lungs were homogenized with a Bullet Blender, then centrifuged at 500 x g for 5 min at 4°C. The supernatant was collected and quick-frozen in ethanol and dry ice and stored at −80°C until the influenza virus plaque assay in Madin-Darwin Canine Kidney MDCK cells was performed. The remaining mice were sacrificed after 14 days to observe the full time-course of influenza virus infection, where body weights and clinical scores were collected daily. For the clinical score, one point was assessed for each of the following traits: piloerection, labored breathing, hunched posture, lethargy, abnormal gait, emaciation (>10% weight loss) for a maximum score of 6. For passive transfer, mice were vaccinated with a booster injection on day 21, then on day 42 vaccinated mice were sacrificed by exsanguination, and approx. 300 μL of serum was collected per mouse. Serum samples were pooled with mice of the same vaccine condition and stored at 4°C prior to transfer. To perform the transfer, 300 μL were introduced to naive mice intraperitoneally via the lower right corner of the abdomen 2 h prior to challenge with H1N1.

Ferret immunization

Ferret care protocols were carried out at Bioqual. Fitch ferrets between 10 and 12 weeks old were immunized intramuscularly on day 0 and 21 with 0.5 mL of hexaplex, or Fluad containing 3 μg of each antigen, or Flublok containing 9 μg of each HA. Sera was collected on day 41 and shipped with dry ice overnight to the University at Buffalo for serological analysis in the same manner that mouse sera was processed.

METHOD DETAILS

Vaccine antigen selection

Recombinant trimeric hemagglutinin and tetrameric neuraminidase for A/Victoria/2570/2019 (H1N1), A/Darwin/6/2021 (H3N2), and B/Austria/1359417/2021 (Victoria lineage) selected for study based on WHO recommendations for trivalent recombinant vaccine formulations for the 2022–2023 influenza season. Further construct information is shown in Figure 1B. They were received in frozen solution then aliquoted and stored at a temperature of −80°C prior to use in vaccines and assays. Samples for use were thawed at room temperature and the remainder was stored at 4°C for up to three weeks between uses. Antigen characterization included SDS-PAGE purity (>95%) and multimerization confirmation by glutaraldehyde crosslinking.

Liposome production

CoPoP/PHAD/QS21 liposomes were synthesized as previously described and were prepared by ethanol injection and nitrogen-pressurized lipid extrusion.[17] The composition of the liposomes included CoPoP, 1,2-dioleoyl-*sn*-glycero-3-phosphocholine (DOPC), cholesterol (PhytoChol), saponin QS21, and synthetic monophosphoryl lipid A Phosphorylated HexaAcyl Disaccharide (PHAD). Lipids were dissolved in 60°C ethanol for 10 min, followed by the addition of 4 mL of 60°C phosphate buffered saline (PBS) for another 10 min at 60°C. Liposomes were then passed through 200, 100 and 80 nm stacked polycarbonate filters in a lipid extruder with nitrogen pressure. After extrusion, liposomes were dialyzed with PBS to remove ethanol. The final liposome concentration was adjusted to a 320 μg/mL stock concentration of CoPoP liposomes and passed through a 0.2 μm sterile filter. Liposome stock was stored at 4°C. The liposome formulation had a mass ratio of [DOPC: CHOL: CoPoP: PHAD: QS21] [4:2:1:0.4:0.4].

Vaccine preparation

Liposomes were incubated with an appropriate quantity of antigen or antigen admixture diluted in sterile PBS to a concentration which allowed for a 1:1 volume incubation mixture of liposome solution and antigen solution. Combined solution was incubated at 37°C for 1 h with gentle shaking to facilitate antigen binding to CoPoP. After binding incubation, the resultant solution was diluted further to the final dosage volume which ensured 50 μL for each animal with ~10% excess. Vaccines were stored at 2°C–8°C overnight prior to vaccination in mice by intramuscular injection (IM). FluBlok is unadjuvanted and its HA recombinant proteins are generated in Sf9 cells, a continuous insect cell line, and Fluad is an inactivated influenza virus vaccine generated in chicken eggs and adjuvanted with MF59 adjuvant. For application in animals, these vaccines were diluted in sterile PBS to appropriate doses based on concentrations in human doses of 45 μg per antigen per 500 μL in Flublok and 15 μg per antigen per 500 μL in Fluad. Flublok and Fluad vaccines were formulated for the 2022–2023 season; recombinant Flublok contained HA antigens from A/Wisconsin/588/2019 (H1N1)pdm09 virus, A/Darwin/6/2021 (H3N2) virus, and B/Austria/1359417/2021 (B/Victoria lineage) virus, while egg-derived Fluad contained both HA and NA antigens from A/Victoria/2570/2019 (H1N1)pdm09 virus, A/Darwin/9/2021 (H3N2) virus, and

B/Austria/1359417/2021 (B/Victoria lineage) virus. These match the hexaplex antigens (considering H1 and N1 from A/Wisconsin/588/2019 are identical to those from A/Victoria/2570/2019). All vaccines were transported from storage to animal facilities on water ice in insulated containers and administered using 0.3 cc insulin needles.

Liposome characterization

Nanoparticle diameters of CoPoP liposomes with bound antigens were determined by dynamic light scattering with a NanoBrook 90 plus PALS instrument after 500-fold dilution in PBS. Nickel nitrilotriacetic acid (Ni-NTA) bead competition binding assay was performed by first incubating His-tagged HA antigens with CoPoP liposomes for 1 h at 37°C. Following this incubation period, Ni-NTA beads were washed 3 times with PBS and separated using a magnetic separator bar. A 25 μL volume of the HA-liposome solution was then introduced to the beads and incubated together for 30 min, with pipetting to resuspend the beads every 10 min. Beads were separated from the solution with the magnetic bar, and the supernatant was re-aliquoted. Beads were resuspended in 25 μL PBS by pipetting. A dye buffer solution was generated by mixing two parts NuPAGE LDS sample buffer (4X) to one part of NuPAGE sample reducing agent (10X). Dye buffer solution was applied in a 1:3 ratio of dye to sample by volume and incubated together at 100°C for 10 min. Beads were then separated from solution by magnetic bar and re-aliquot, generating the bead fraction. Sample channels were loaded with a 25 μL volume in a stain-free NuPAGE gel alongside 10 μL of a protein molecular weight ladder. The gel was run at a voltage of 200 V for 45 min, washed once, and then the gel was imaged using a GelDoc Go Imaging System.

Cryo-transmission electron microscopy characterization

Samples analyzed by cryo-TEM were vitrified using a Vitrobot Mark IV. To increase the concentration of liposomes inside the holes in the grids, samples were applied twice to the grids. In the first application a volume of 3.6 μL was applied to the holey carbon grids and manually blotted using the Vitrobot blotting paper (Standard Vitrobot Filter Paper, Ø55/20mm, Grade 595). Right after blotting, a new drop of the sample was applied to the EM grid and blotted again using the standard routine with the two blotting pads in the Vitrobot Mark IV for 3 s and with a blot force +1 before they were plunged into liquid ethane. The Vitrobot was set at 25°C and 100% relative humidity. The grids used for these experiments were C-flat 2/2-2Cu-T and before the samples were applied, they were washed with chloroform for 2 h and treated with negative glow discharge in air at 5mA for 15 s.

Data was acquired using SerialEM software on the Titan Krios electron microscope at FEMR-McGill, operated at 300kV. The sample was kept at −190°C during imaging. Images were collected with a Gatan K3 direct electron detector equipped with a Bioquantum imaging filter at defocuses between −2.00 to −4.00 μm and total exposure of 50 $e-/Å^2$. We used a nominal magnification of 81,000 × corresponding to images with a calibrated pixel size of 1.09 Å.

Slot blot

A 0.2 μm nitrocellulose membrane was pre-wet in PBS and set onto a 48-well slot blot apparatus with wells filled with 10ng H1, H3, HB, N1, N2, NB, or a mixture of these six proteins incubated with liposomes. Fifty microliters of PBS were loaded into the wells to investigate any leakage. Samples were then loaded into the wells and flew through the membrane for 10-min. The membrane was cut into strips and blocked using 5% BSA in PBS for 1 h at RT followed by washing with PBS twice. The membrane was incubated with diluted mouse monoclonal antibodies in 5% BSA/PBS on a plate shaker for 1 h at RT. Membrane strips were washed with PBS for three times, followed by incubation with HRP-linked secondary anti-mouse antibody at 1:2000 dilution in 5% BSA/PBS for 30 min at RT. The strips were treated with VisiGlo HRP substrate mixture after washing with PBS three times and imaged using a Bio-Rad ChemiDoc Imager.

Serological antibody ELISA

Vaccinated mouse serum with an initial 200-fold dilution in PBS with 0.1% Tween 20 was subjected to 10-fold serial dilution to a maximum dilution factor of 1.0 x 10^7. ELISA plates were coated with 100 μL/well of 1 μg/mL antigen in coating buffer (5.3 g/L NA_2CO_3 and 4.2 g/L $NAHCO_3$ in distilled water, pH 9.6) and incubated overnight, at least 18 h. Plates were then washed 3 times and incubated with 1% bovine serum albumin (BSA) for 2 h at 37°C. BSA solution was removed, and 100 μL of serum dilutions were added, followed by a 1-h incubation at 37°C. Plates were washed 3 times and 100 μL of 1 μg/mL of goat anti-mouse secondary antibody with HRP was added and incubated an additional 30 min at 37°C. Plates were then washed 6 times and 100 μL of 3,3′,5,5′-Tetramethylbenzidine (TMB) solution was added and allowed to develop for 5 min. 100 μL of HCl was used to stop the reaction and absorbance was read at 450 nm.

Serological antibody inhibition and neutralization

Serum samples were prepared by combining 20 μL of serum with 60 μL of receptor destroying enzyme (RDE II) and incubating at 37°C for 18 h. Temperature was increased to 56°C for 30 min to allow for heat-inactivation of enzymes before dilution to a final volume of 200 μL with sterile PBS, resulting in an initial 10-fold dilution of serum. Samples were transferred to 96-well plates and 2-fold serial dilutions were performed in PBS to form a dilution range from 1/10 to 1/1280. Dilution curves were generated to find the 4 hemagglutinating unit (HAU) concentration of mouse-adapted A/California/04/2009 H1N1, A/Hong Kong/1/1968 H3N2, and B/Malaysia/2506/2004. Equal parts serum and virus dilutions were incubated together for 1 h at 37°C with steady shaking, then a 1% turkey red blood cell suspension in sterile PBS was added and allowed to incubate at room temperature for 30 min. HAI titer

was determined by highest dilution with complete inhibition of hemagglutination, as determined by comparison against virus-free reference control wells.

Neuraminidase inhibition measurements were performed using the enzyme-linked lectin assay (ELLA) assay method. Nunc 96-well ELISA plates were incubated 24 h at 4°C with 25 μg/mL fetuin in PBS. Viral stocks were diluted in coated test plates to determine the absorbance curve and identify the ideal viral stock dilution to achieve 90% absorbance on the linear phase of the curve. Serum samples underwent serial dilution in an uncoated plate, then diluted viral stocks were added and samples were incubated for 1 h at 37°C with steady shaking to allow antibody-NA interactions prior to fetuin exposure. Samples were then transferred to a coated plate and incubated at 37°C for 18 h. After incubation plates were washed 6 times with 100 μL PBST and incubated with 1 μg/mL PNA-HRP in PBST for 2 h at room temperature under dark conditions. Plates were then washed again 3 times with PBST and treated with 100 μL TMB and allowed to develop for 10 min. 100 μL of HCl was used to stop the reaction and absorbance was read at 450 nm.

ELISPOT

Spleens were excised and immediately placed in 1 mL ice-cold PBS. Cell strainers with 70 μm mesh were wetted with PBS and used to separate splenocytes from pulverized spleen tissue. The resulting suspension of cells in PBS was centrifuged at 500 rcf for 5 min, then supernatant was decanted, and 3 mL of RBC lysis buffer was added. Following 10 min incubation on ice, 10 mL of PBS was added, and suspensions were centrifuged again at 500 rcf for 5 min and supernatant decanted. Cells were finally resuspended in 1 mL of 0.1% BSA, 0.1% sodium azide solution in PBS (wash buffer). Cells were counted using an EVE automatic cell counter. A total of 3 × 105 splenocytes or lung cells, were seeded in an ELISpot plate, and 5 μg/mL of RBD was added to each well. The cells were cultured in 5% CO_2/95% air at 37°C in a humidified chamber for 24 h. The detection of spots was performed according to the manufacturer instructions from Immunospot, using the Murine IFN-γ Single-Color ELISPOT kit. The next day, the plate was washed twice with PBS and twice with PBS-T. The wells were incubated with 80 μL of anti-murine IFNγ antibody detection buffer for 2 h. Later, the wells were washed three times with PBS-T, then incubated with Streptavidin-AP for 0.5 h. After the incubation, each well was washed with PBS-T twice and distilled water twice. To develop spots, the plates were incubated for 15 min at RT, with 80 μL per well of blue developer solution provided from the manufacturer. The images were acquired with CTL ImmunoSpot S6 FluoroCore analyzer.

Influenza virus plaque assay in MDCK cells

MDCK cells were grown to 70 to 80% confluency in DMEM +0.1mM nonessential amino acids + 1mM sodium pyruvate +50 U/mL penicillin and 50 μg/mL streptomycin +20 μg/mL gentamycin +10% fcs in 6-well tissue culture plates in 37°C + 5% CO_2. The medium was removed, and the cells rinsed twice with 3% BSA in DMEM, then 100 μL 10-fold serial dilutions (3% BSA in DMEM as diluent) of virus samples were adsorbed for 1 h at 37°C + 5% CO_2 (300 μL diluent was added to each well during the adsorption to prevent drying of cell sheet). The inoculum was removed, the cells rinsed once with PBS, and then overlaid with 2 mL L-15 medium +1 μg/mL TPCK-treated trypsin +0.8% agarose. The plates were incubated for 48 h then fixed in 90% ethanol for 30 min and stained with 0.3% crystal violet +5% isopropanol +5% ethanol in distilled water for 20 min following removal of the overlay. The cells were rinsed with distilled water and the plaques enumerated.

QUANTIFICATION AND STATISTICAL ANALYSIS

All statistical analysis was performed using GraphPad Prism 9 software. Data in figures is presented as the mean ± standard error of the mean (SEM). Means are calculated on the value of n biological replicates, with n dependent upon experiment goals; n = 5 for serological analysis, n = 9 for mouse challenge studies for the first 4 days, then n = 3 in lung viral assays and n = 6 for survival challenge. Statistical analysis was calculated using GraphPad Prism 9 software functions, with settings for one-way ANOVA performed with Tukey's multiple comparisons, or log rank test for the statistical analysis of survival curves.

Cell Reports Medicine, Volume *5*

Supplemental information

Adjuvanted nanoliposomes displaying six hemagglutinins and neuraminidases as an influenza virus vaccine

Zachary R. Sia, Jayishnu Roy, Wei-Chiao Huang, Yiting Song, Shiqi Zhou, Yuan Luo, Qinzhe Li, Dominic Arpin, Hilliard L. Kutscher, Joaquin Ortega, Bruce A. Davidson, and Jonathan F. Lovell

Supporting Information: Adjuvanted Nanoliposomes Displaying Six Hemagglutinins and Neuraminidases for an Influenza Virus Vaccine

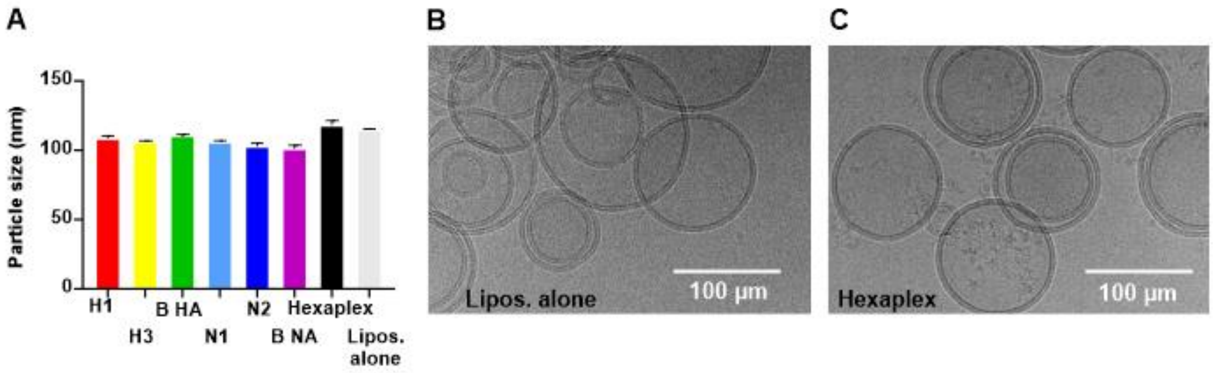

Figure S1. Particle Size Measurements. (A) Particle size is consistent between undecorated, monovalent and hexaplex liposomes. (B & C) Liposome morphology observed by cryo-TEM shows intact liposome morphology. (C) Some proteins can be observed in the objective focus on the liposome surface. Related to Figure 1. Particle size technical replicates of n = 3 were used.

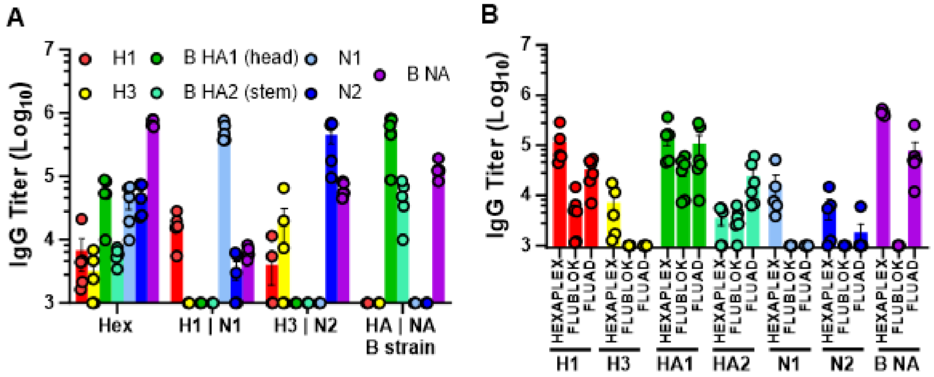

Figure S2. Antigen Cross-Reactivity Without Multimerization Domains. (A) Serum antibodies were tested against heterologous antigens without polymerization domain. These strains differed from those in figure 2; included strains were A/Wisconsin/588/2019 (H1N1), A/Kansas/14/2017 (H3N2), and B/Brisbane/60/2008. (B) Compared to Flublok and Fluad, hexaplex produced the greatest quantity of cross-reactive IgG antibodies not specific to the polymerization domains. Biological replicates of n = 5 were used. This data supplements information in the main text Figure 2 and 4.

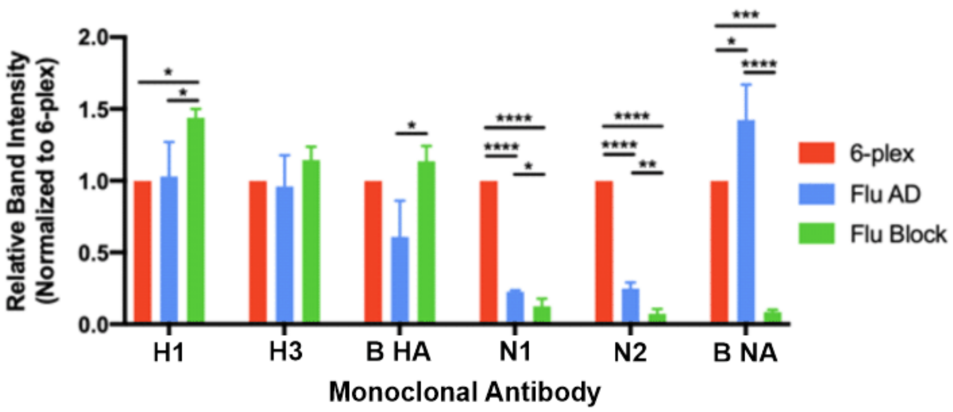

Figure S3. Slot Blot Band Quantification. Band intensity for slot blot in figure 4B, normalized to the bands presented by the hexaplex nanoliposomes. Statistical analysis was performed by one-way ANOVA with Tukey's post-hoc multiple comparisons. Markers indicate *p<0.05, **p<0.01, ***p<0.005, ****p<0.001. Related to Figure 4.

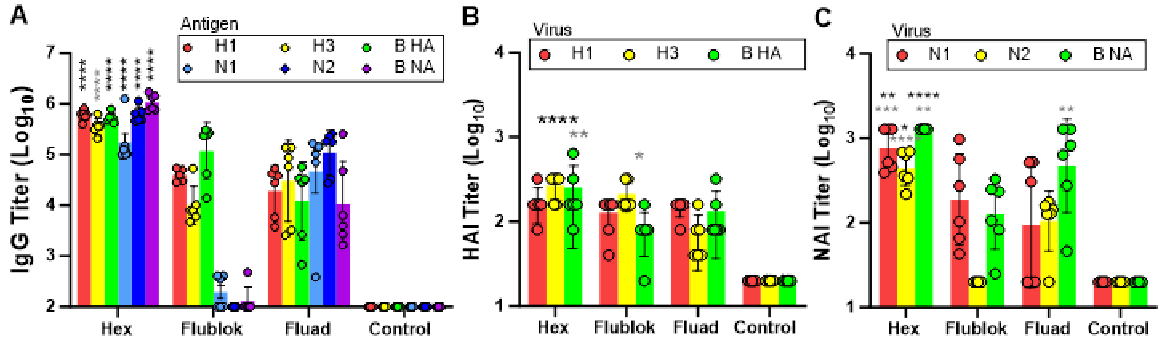

Figure S4. Additional Serological Analysis of Vaccine in Mice. Serum from mice vaccinated with hexaplex or comparator vaccines were assessed in serological assays against viruses A/California/04/2009 (H1N1), A/New York/39/2012 (H3N2), and B/Washington/02/2019 (Victoria lineage). (A) Hexaplex yielded significantly higher IgG titers than either Flublok or Fluad. (B) HAI titers were comparable among vaccines, with hexaplex yielding significant advantage over Fluad. (C) NAI was significantly higher in hexaplex vaccinated mouse serum than either Flublok or Fluad. Statistical analysis was performed with two-way ANOVA with multiple comparisons. Markers indicate *p<0.05, **p<0.01, ***p<0.005, ****p<0.001, black markers indicate significance relative to Fluad, while grey markers indicate significance relative to Flublok. Biological replicates of n = 5 were used. Related to Figure 4.

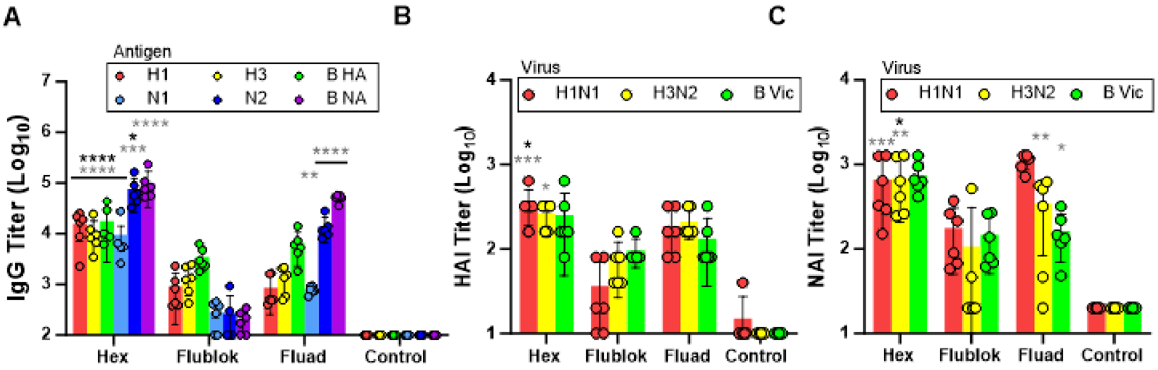

Figure S5. Serological Analysis of Hexaplex Vaccine in Ferrets. Serum from ferrets vaccinated with hexaplex, flublok, and fluad, were assessed using serological assays as previously described. (A) Hexaplex vaccine elicited highest overall specific binding antibody titers, with Fluad achieving comparable titers against B NA. (B) Hemagglutination inhibition titers were comparable between hexaplex and fluad vaccines, with hexaplex vaccine yielding significantly higher HAI against H1N1. (C) Neuraminidase inhibition was likewise comparable to Fluad, with superior NAI against B Victoria lineage virus. Asterisks indicate significance relative to hexaplex; asterisks over hexaplex bars indicate significant increase over all other groups, while asterisks over comparator bars indicates significant increase over hexaplex. Statistical analysis was performed with two-way ANOVA with multiple comparisons. Markers indicate *$p<0.05$, **$p<0.01$, ***$p<0.005$, ****$p<0.001$, black markers indicate significance relative to Fluad, while grey markers indicate significance relative to Flublok. Biological replicates of n = 6 were used. Related to Figure 5.

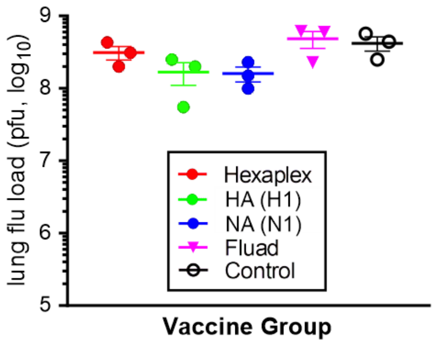

Figure S6. Lung Viral Load During Passive Transfer Challenge. Virus load in the homogenized lung tissue of mice was measured on day 4 post-infection by euthanasia and excision from 3 mice within the initial groups. Statistical analysis by one-way ANOVA identified no significant differences between groups. Biological replicates of n = 3 were used. Related to Figure 6.

% Identity with Vaccine Strains

	HA			NA		
**Virus Strain**	A/Victoria /2570/2019 (H1N1)	A/Darwin/ 6/2021 (H3N2)	B/Austria/ 1359417/2 021 (Victoria)	A/Victoria/ 2570/2019 (H1N1)	A/Darwin/ 6/2021 (H3N2)	B/Austria/ 1359417/2021 (Victoria)
H1N1						
A/California/04/2009	95.05	41.70	29.26	94.46	43.07	35.46
H3N2						
A/Hong Kong/1/1968	45.39	84.28	26.97	41.93	84.22	29.85
B Victoria						
B/Malaysia/2506/2004	28.95	27.41	97.09	31.93	32.37	95.49
B Yamagata						
B/Phuket/3073/2013	28.95	28.25	92.12	32.42	28.51	94.42

Table S1. Protein Sequence Identity by Protein BLAST. Antigen sequences acquired from the GISAID database were compared by Protein BLAST multiple sequence comparison, with percentage identity of alignments for both HA and NA antigens compared by strain here. Related to Figure 3
